# Human-induced pluripotent stem cell–based hepatic modeling of lipid metabolism–associated TM6SF2-E167K variant

**DOI:** 10.1097/HEP.0000000000001065

**Published:** 2024-08-27

**Authors:** Lanuza A.P. Faccioli, Yiyue Sun, Olamide Animasahun, Takashi Motomura, Zhenghao Liu, Takeshi Kurihara, Zhiping Hu, Bo Yang, Zeliha Cetin, Annalisa M. Baratta, Ajay Shankaran, Minal Nenwani, Leyla Nurcihan Altay, Linqi Huang, Noah Meurs, Jonathan Franks, Donna Stolz, Dillon C. Gavlock, Mark T. Miedel, Alina Ostrowska, Rodrigo M. Florentino, Ira J. Fox, Deepak Nagrath, Alejandro Soto-Gutierrez

**Affiliations:** 1 Department of Pathology, University of Pittsburgh, Pittsburgh, Pennsylvania, USA; 2 Department of Pathology, Center for Transcriptional Medicine, University of Pittsburgh, Pittsburgh, Pennsylvania, USA; 3 Department of Pathology, Pittsburgh Liver Research Center, University of Pittsburgh, Pittsburgh, Pennsylvania, USA; 4 School of Medicine, Tsinghua University, Beijing, PRC; 5 Department of Chemical Engineering, University of Michigan, Ann Arbor, Michigan, USA; 6 Department of Biomedical Engineering, Biointerfaces Institute, University of Michigan, Ann Arbor, Michigan, USA; 7 Laboratory for Systems Biology of Human Diseases, University of Michigan, Ann Arbor, Michigan, USA; 8 Department of Biomedical Engineering, University of Michigan, Ann Arbor, Michigan, USA; 9 Department of Cell Biology and Physiology, Center for Biologic Imaging, University of Pittsburgh, Pittsburgh, Pennsylvania, USA; 10 Drug Discovery Institute, Department of Computational and Systems Biology, University of Pittsburgh, Pittsburgh, Pennsylvania, USA; 11 Department of Surgery, Children’s Hospital of Pittsburgh of UPMC, University of Pittsburgh, Pittsburgh, Pennsylvania, USA

**Keywords:** human hepatocytes, TM6SF2 E167K, iPSC, liver disease model, lipid metabolism

## Abstract

**Background and Aims:**

TM6SF2 rs58542926 (E167K) is related to an increased prevalence of metabolic dysfunction–associated steatotic liver disease. Conflicting mouse study results highlight the need for a human model to understand this mutation’s impact. This study aims to create and characterize a reliable human in vitro model to mimic the effects of the TM6SF2-E167K mutation for future studies.

**Approach and Results:**

We used gene editing on human-induced pluripotent stem cells from a healthy individual to create cells with the TM6SF2-E167K mutation. After hepatocyte-directed differentiation, we observed decreased TM6SF2 protein expression, increased intracellular lipid droplets, and total cholesterol, in addition to reduced VLDL secretion. Transcriptomics revealed the upregulation of genes involved in lipid, fatty acid, and cholesterol transport, flux, and oxidation. Global lipidomics showed increased lipid classes associated with endoplasmic reticulum (ER) stress, mitochondrial dysfunction, apoptosis, and lipid metabolism. In addition, the TM6SF2-E167K mutation conferred a proinflammatory phenotype with signs of mitochondria and ER stress. Importantly, by facilitating protein folding within the ER of hepatocytes carrying TM6SF2-E167K mutation, VLDL secretion and ER stress markers improved.

**Conclusions:**

Our findings indicate that induced hepatocytes generated from human-induced pluripotent stem cells carrying the TM6SF2-E167K recapitulate the effects observed in human hepatocytes from individuals with the TM6SF2 mutation. This study characterizes an in vitro model that can be used as a platform to identify potential clinical targets and highlights the therapeutic potential of targeting protein misfolding to alleviate ER stress and mitigate the detrimental effects of the TM6SF2-E167K mutation on hepatic lipid metabolism.

## Graphical Abstract

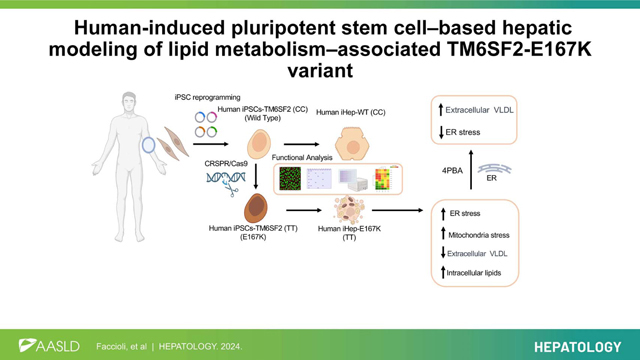

## INTRODUCTION

Chronic liver disease (CLD) progressing to liver failure, HCC, and portal hypertension result in 2 million deaths annually worldwide. HCC alone ranks as the fifth leading cause of cancer-related deaths in the United States.^[1,2]^ The etiology of CLD has changed dramatically over the last few decades.^[3]^ The burden of chronic hepatitis C has diminished due to the emergence of highly effective direct-acting antiviral agents, while the incidence of metabolic dysfunction–associated steatotic liver disease (MASLD), including metabolic dysfunction–associated steatohepatitis (MASH), has grown. MASLD is currently the leading indication of adult liver transplantation and significantly increases an individual’s risk for developing HCC and associated CLD.^[4–8]^ Despite its public health importance and financial burden, there is currently no FDA-approved therapy for MASLD. The lack of therapeutic options reflects the complex patient pathogenesis and heterogeneity, as well as the lack of experimental models that fully recapitulate disease phenotypes. Thus, our current ability to predict disease progression and response to treatments is limited.

Genome-wide association studies have identified numerous genetic variants^[9]^ associated with MASLD. These include single nucleotide polymorphisms in PNPLA3 (rs738409 C > G p.Ie148Met; patatin-like phospholipase domain-containing protein 3), MBOAT7 (rs62641738 C > T; membrane bound *O*-acyltransferase domain-containing 7), GCKR (rs780094 C > T; glucokinase regulator), and TM6SF2 (rs58542926 C > T p.Glu167Lys; transmembrane 6 superfamily 2).^[9–12]^ This missense variant in *TM6SF2* causes the substitution of glutamine with a lysine at position 167 (p. Glu167Lys or E167K) and leads to protein misfolding with concomitant accelerated protein degradation,^[10]^ an increase in serum cholesterol levels, and accumulation of lipids in the liver. Individuals carrying the TM6SF2-E167K variant exhibit a 2-fold higher prevalence of both MASH and advanced fibrosis^[10,13]^ and are at increased risk for liver transplantation and liver-related death.^[14]^ As this variant is rare,^[15]^ the precise molecular mechanism by which this mutation influences the development of liver disease has not been fully elucidated.^[9,10,16]^

Although there is a substantial and continually growing body of literature describing the role of TM6SF2 in lipid accumulation and increasing cholesterol levels, the study of this phenomenon in loss-of-function or overexpression mouse models has generated inconsistent findings.^[17–19]^ Therefore, the creation of a human model that can illuminate the effects of this variant is of great value for MASLD studies and therapeutic development. Thus, we generated human-induced pluripotent stem cells (iPSCs) from an individual who carried the WT *TM6SF2* allele (CC) and, using CRISPR-Cas9, generated gene-edited iPSCs carrying the *TM6SF2* mutant allele (TT). These iPSC lines were differentiated into hepatocytes (iHeps).^[20]^ Characterization of these cell lines revealed expression differences of TM6SF2 protein and accumulation of triglycerides, apolipoprotein B100 (ApoB100), and total cholesterol with decreased secretion of VLDL. We dissected the molecular consequences of the TM6SF2-E167K variant using transcriptomics and unveiled alterations related to the transport, flow, removal, and oxidation of lipids, fatty acids, and cholesterol. Finally, we observed that treating cells with a drug that reduces endoplasmic reticulum (ER) stress normalized VLDL secretion and ER stress. Collectively, our results suggest that this human model can be used to replicate and study the role of genetic variants in the development of CLD. In addition, it highlights the therapeutic potential of targeting protein misfolding to alleviate ER stress and mitigate the detrimental effects of the TM6SF2-E167K mutation on hepatic lipid metabolism.

## METHODS

### Generation and culture of human iPSC

iPSC-TM6SF2-WT was generated from fibroblasts. Fibroblasts were reprogrammed using episomal plasmid vectors adapted from a previously described method.^[21]^ See Supplemental Materials and Methods for details, http://links.lww.com/HEP/I640.

### Gene editing

The single-guide RNA sequence (GCAAATACAGCTCCGAGATC) was designed to cut the human TM6SF2 gene at position chr19:379,549 and replace the major allele (C) with the minor allele (T). See Supplemental Materials and Methods for details, http://links.lww.com/HEP/I640.

### Differentiation of human iPSCs into induced hepatocytes (iHep)

Our hepatocyte differentiation protocol was reported by de l’Hortet et al.^[21]^ See Supplemental Materials and Methods for details, http://links.lww.com/HEP/I640.

### RNA-seq, differential gene analysis, and gene set enrichment analysis

Whole-genome strand-specific RNA-seq was used to profile RNA expression levels in iHep-TM6SF2-WT and iHep-TM6SF2-E167K. RNA-seq libraries were prepared as described.^[21]^ Comparisons to population data were performed using the Kyoto Encyclopedia of Genes and Genomes Pathway Enrichment Analysis and over-representation analysis algorithm. The significantly altered pathways were compared to those identified from the supplementary data of Prill et al.^[22]^ These results were visualized with the R software (v.4.2.3) package ggplot2 (v.3.4.2). See Supplemental Materials and Methods for details, http://links.lww.com/HEP/I640. RNA-seq raw data for iPSC TM6SF2 WT and E167K have been deposited with links to BioProject: PRJNA1055672 in the NCBI BioProject database (https://www.ncbi.nlm.nih.gov/bioproject/).

### Lipidomics

For analysis of lipidomics, iHep-TM6SF2-WT and iHep-TM6SF2-E167K cells were collected. The sample extracts were analyzed using an LC-ESI-MS/MS system (UPLC, Nexera LC-40; MS, Triple Quad 6500+). Differential analysis was performed using MetwareBio’s bioinformatics pipeline. See Supplemental Materials and Methods for details, http://links.lww.com/HEP/I640.

### 4-Phenylbutyric acid treatment

4-Phenylbutyric acid (4PBA) was freshly prepared before each experiment by dissolving 4PBA powder (Sigma Aldrich) in PBS to a final concentration of 27 mM. This stock was later diluted in PBS and used at a final concentration of 2 mM. Cells were treated for 48 hours, and a control group without 4PBA treatment was maintained to compare the effects. After 48 hours, supernatant and cell pellets were collected for further analysis.

### Primary human hepatocytes, embryoid body formation, quantitative real-time PCR, genotyping and Sanger sequencing, immunostaining, ELISA, transcription profiling by the RT2 Profiler PCR array, fatty acid synthesis assay, Nile Red staining, cholesterol analysis, western blotting, transmission electron microscopy, caspase assay, reactive oxygen species assay, total NAD/NADH, quantification, insulin-resistance response, human cytokine antibody array, fatty acid uptake assay, statistical analysis

See Supplemental Materials and Methods for details, http://links.lww.com/HEP/I640 and Supplemental Table S1, http://links.lww.com/HEP/I672, Supplemental Table S2, http://links.lww.com/HEP/I673 and Supplemental Table S3, http://links.lww.com/HEP/I674.

## RESULTS

### Generation of human iPSCs and introduction of the TM6SF2-E167K variant

**FIGURE 1:**
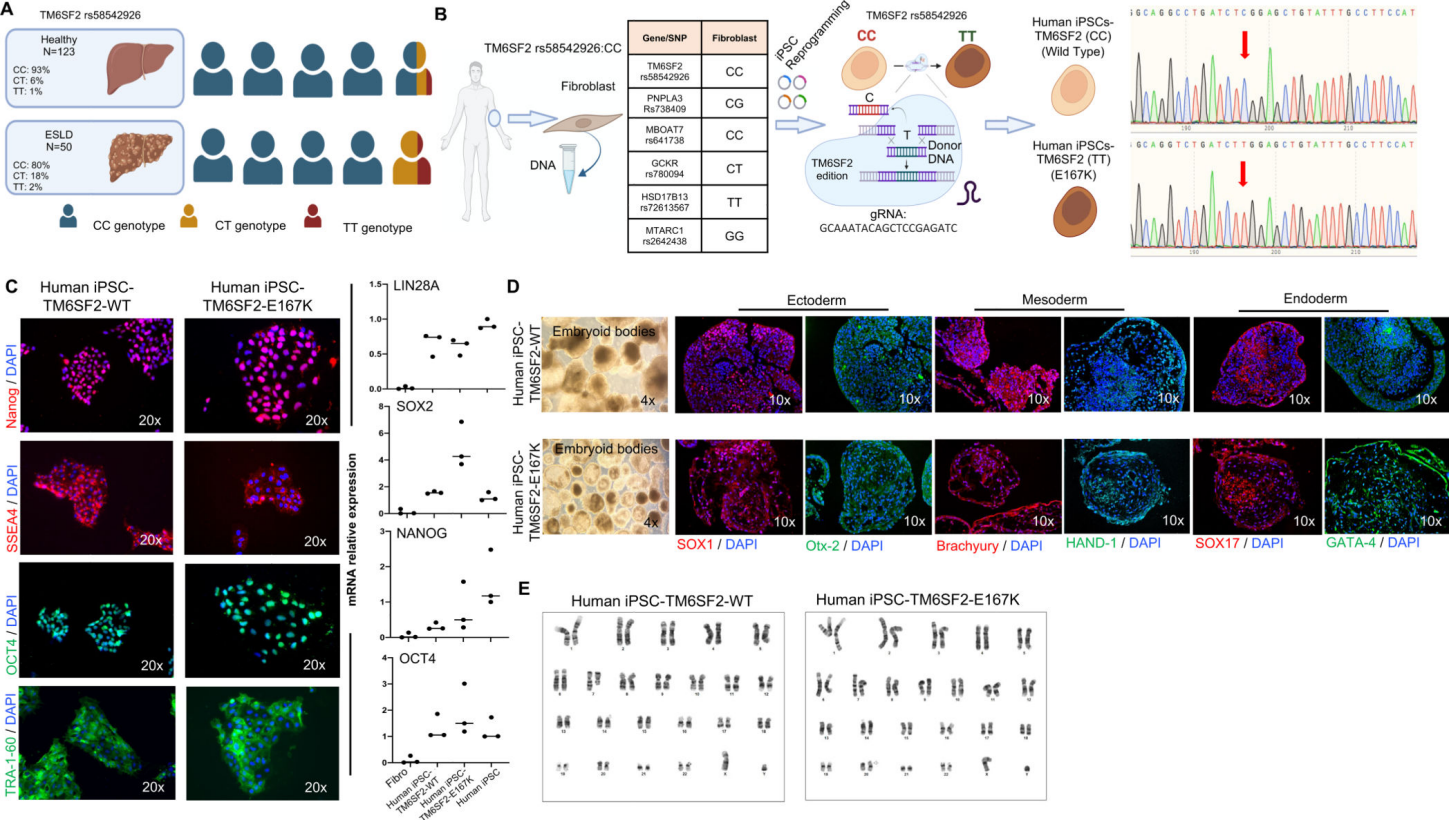
Generation and characterization of iPSC-TM6SF2-WT and iPSC-TM6SF2-E167K. (A) Genotype frequency of the *TM6SF2* rs58542926 variant in a US cohort (healthy individuals, n = 123, and ESLD samples, n = 50). Human symbols represent 20% of the prevalence. (B) Schematic design of the generation of iPSC-TM6SF2-WT and iPSC-TM6SF2-E167K. We generated iPSC-TM6SF2-WT from fibroblasts obtained from a healthy individual, followed by gene editing using CRISPR/Cas9 to generate iPSC-TM6SF2-E167K. Sanger sequencing confirmed that iPSC-TM6SF2-WT cells were major homozygous (CC) and iPSC-TM6SF2-E167K cells are minor homozygous (TT) after gene editing for the TM6SF2 rs58542926, as indicated by the red arrow. (C) Immunofluorescence micrographs of pluripotency markers: Nanog, SSEA4, OCT4, and TRA-1–60 (left panel) and quantitative gene expressions of pluripotency markers: SOX2, LIN28A, OCT4, and Nanog (right panel) in both iPSC-TM6SF2-WT (n = 3) and iPSC-TM6SF2-E167K (n = 3). WTC11 cells were used as a positive control, and human fibroblasts were used as a negative control. Values are determined relative to β-actin and presented as fold change relative to the expression in human WTC11, which is set as 1. (D) Micrographs of embryoid bodies and immunofluorescence micrographs of the three germ layer markers: ectoderm (SOX1 and OTX-2), mesoderm (HAND-1 and Brachyury), and endoderm (SOX17 and GATA-4) in both iPSC-TM6SF2-WT and iPSC-TM6SF2-E167K. (E) G-banding analysis for karyotype in both iPSC-TM6SF2-WT and iPSC-TM6SF2-E167K shows no abnormalities in the cells. Abbreviation: iPSC, induced pluripotent stem cell.

First, we assessed the incidence of TM6SF2 rs58542926 C > T p.Glu167Lys frequency in healthy subjects and patients with ESLD.^[13]^ We focused on the TM6SF2 rs58542926 C > T variant in donors and explanted human cirrhotic livers with ESLD due to MASH, as robust genome-wide association studies have linked this variant to a spectrum of liver diseases, as well as increased risk of mortality in the general population. The TM6SF2-E167K variant was present in < 1% of healthy individuals and 1.8% of patients with MASH-associated ESLD (Figure 1A and Supplemental Table S5, http://links.lww.com/HEP/I634). This analysis shows that the TM6SF2-E167K variant was present at a low frequency in the small (healthy, n = 123; ESLD, n = 50) cohort analyzed,^[13]^ and its frequency was maintained in ESLD.^[16,17]^

Next, we identified human fibroblasts carrying the major allele TM6SF2 rs58542926:C without the presence of other variants predictive of liver disease (MBOAT7 rs641738, TM6SF2 rs58542926, GCKR rs780094, HSD17B13 rs72613567, and MTARC1 rs2642438; Figure 1B). After genotyping, human fibroblasts were reprogrammed into iPSCs as described.^[20,21]^ The resulting human iPSC line (iPSC-TM6SF2-WT) was single nucleotide edited using CRISPR-Cas9 to carry the TM6SF2-E167K variant (iPSC-TM6SF2-E167K). The resulting iPSCs (iPSC-TM6SF2-WT and iPSC-TM6SF2-E167K) were cultured for > 10 passages before characterization and validation studies were performed. Successful single base editing was confirmed by Sanger sequencing and showed the presence of the gene variant for *TM6SF2* rs58542926 C > T (Figure 1B). To evaluate the potential off-target effects of the designed single-guide RNA, cleavage efficiency targeting the TM6SF2 locus was assessed in HepG2 cells, and it was found that mutations were specifically induced at the targeted site. In addition, to further validate that there were no off-target effects, iPSC-TM6SF2-E167K were sequenced upstream and downstream, and no off-target effects were observed in either the gRNA (Supplemental Figure S1A, http://links.lww.com/HEP/I635) or iPSC-TM6SF2-E167K (Supplemental Figure S1B, http://links.lww.com/HEP/I635).

Human iPSC-TM6SF2-WT and iPSC-TM6SF2-E167K showed normal pluripotent morphology, consisting of compact colonies with distinct borders, as seen in human embryonic stem cells, expressed NANOG, SSEA4, OCT4, and TRA-1–60, and exhibited mRNA expression of pluripotency markers (Lin28A, SOX2, Nanog, and OCT4) comparable to that of control human iPSCs (Figure 1C). EBs derived from human iPSC-TM6SF2-WT and iPSC-TM6SF2-E167K lines formed all 3 germ layers (Figure 1D), as assessed by the spontaneous expression of ectodermal (SOX1 and Otx-2), mesodermal (Brachyury and HAND-1), and endodermal (SOX17 and GATA-4) markers (Figure 1D). Both human iPSC-TM6SF2-WT and iPSC-TM6SF2-E167K cells exhibited a normal karyotype (Figure 1E).

### Hepatocyte-directed differentiation of human iPSC-TM6SF2-WT and iPSC-TM6SF2-E167K

**FIGURE 2:**
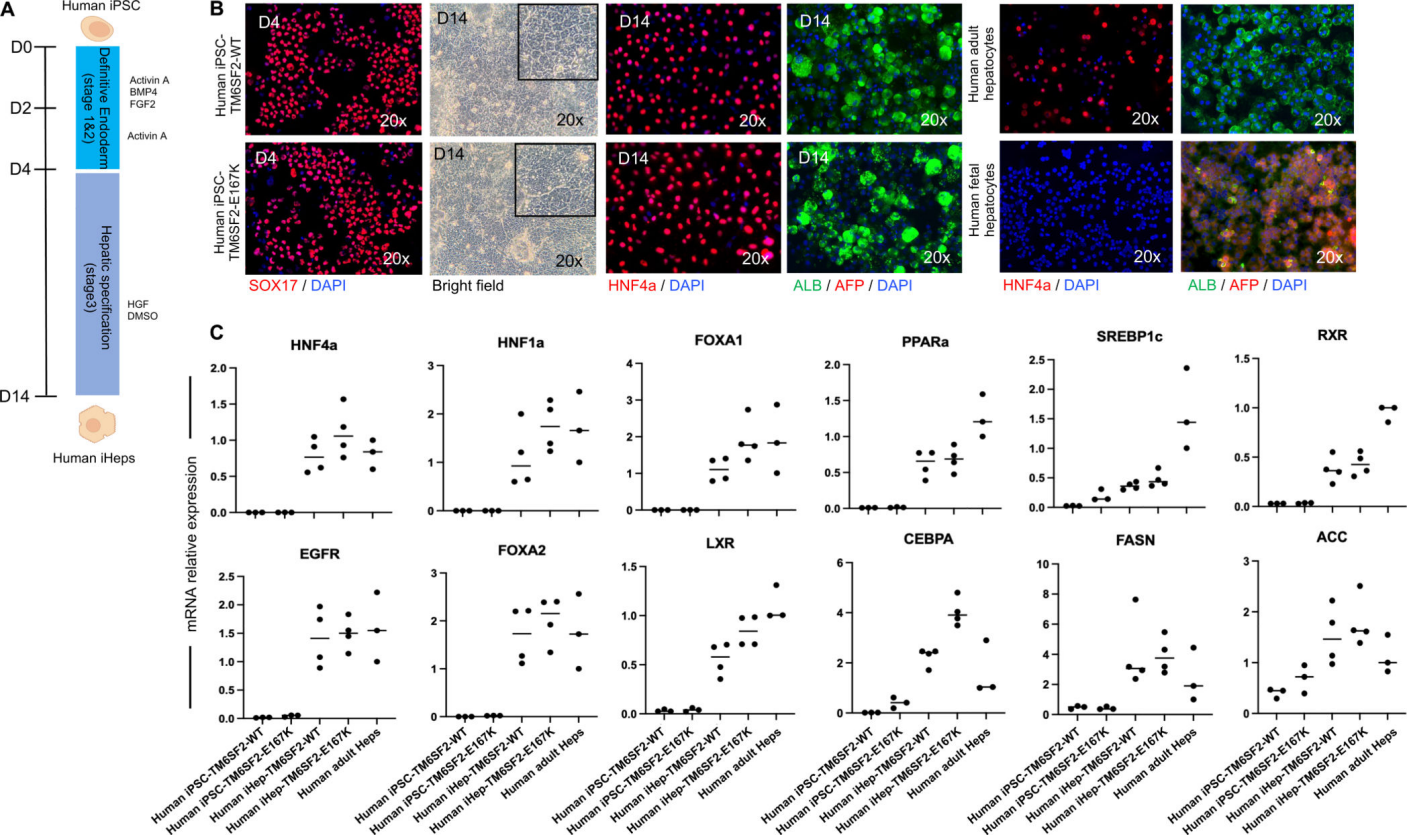
Hepatic differentiation and characterization of iHep-TM6SF2-WT and iHep-TM6SF2-E167K. (A) Schematic illustration of the hepatocyte differentiation protocol, highlighting the 3 main stages of differentiation by sequential addition of defined medium protocols containing Activin-A, BMP-4, and FGF2 (stage 1); Activin-A (stage 2); and DMSO and HGF (stage 3). (B) Immunofluorescence micrographs (left panel) of endoderm marker SOX17 in both iDE-TM6SF2-WT (n = 4) and iDE-TM6SF2-E167K (n = 4). Bright-field micrographs of iHep-TM6SF2-WT and iHep-TM6SF2-E167K show cells on the last day of stage 3. Immunofluorescence micrographs of hepatocyte markers, adult isoform HNF4α, AFP, and albumin in both iHep-TM6SF2-WT and iHep-TM6SF2-E167K. Human adult hepatocytes (PHH) (n = 3) and human fetal hepatocytes (n = 3) were used as positive and negative controls, respectively. (C) Quantitative gene expression for hepatocyte markers: HNF4α, HNF1α, FOXA1, FOXA2, PPARα, LXR, RXR, FASN, EGFR, SREBP1c, ACC, and CEBPA in both iHep-TM6SF2-WT (n = 4) and iHep-TM6SF2-E167K (n = 4). PHH cells (n = 3) were used as a positive control, and both undifferentiated iPSC lines (n = 3) were used as a negative control. Values are determined relative to β-actin and presented as fold change relative to the expression in PHH, which is set as 1. Abbreviations: ACC, acetyl-CoA carboxylase; CEBPA, CCAAT enhancer binding protein alpha; EGFR, epidermal growth factor receptor; FASN, fatty acid synthase; FOXA1, forkhead box protein A1; FOXA2, forkhead box protein A2; HNF1α, hepatocyte nuclear factor 1 alpha; LXR, liver X receptor; PPARα, peroxisome proliferator-activated receptor alpha; RXR, retinoid X receptor; SREBP1c, sterol regulatory element-binding transcription factor 1.

We proceeded to differentiate the human iPSC-TM6SF2-WT and iPSC-TM6SF2-E167K toward hepatocytes using our previously published protocol (Figure 2A).^[20,21,23,24]^ Cells were cultured with a combination of activin A, bone morphogenetic protein 4, and FGF-2 to induce definitive endoderm. After verifying the presence of the endoderm marker SOX17 (Figure 2B), cells were cultured for 10 days in the presence of DMSO and human HGF to induce hepatocyte specificity. Following differentiation, both human iPSC-TM6SF2-WT and iPSC-TM6SF2-E167K developed characteristics of hepatocytes, including expression of the adult isoform of HNF4α and human albumin. Expression of AFP, an immature hepatocyte marker, was not observed (Figure 2B). Both cell lines (human iHeps-TM6SF2-WT and iHeps-TM6SF2-E167K) expressed critical hepatocyte-specific transcripts, including HNF4α, forkhead box protein A2, forkhead box protein A1, hepatocyte nuclear factor 1 alpha, CCAAT enhancer binding protein alpha, retinoid X receptor, liver X receptor, peroxisome proliferator-activated receptor alpha, sterol regulatory element-binding transcription factor 1 (SREBP1c), acetyl-CoA carboxylase (ACC), fatty acid synthase (FASN), and EGF receptor, at levels comparable to those found in human adult hepatocytes (Figure 2C).

### TM6SF2-E167K variant induces protein loss-of-function and modifies lipid accumulation in human iHeps

**FIGURE 3:**
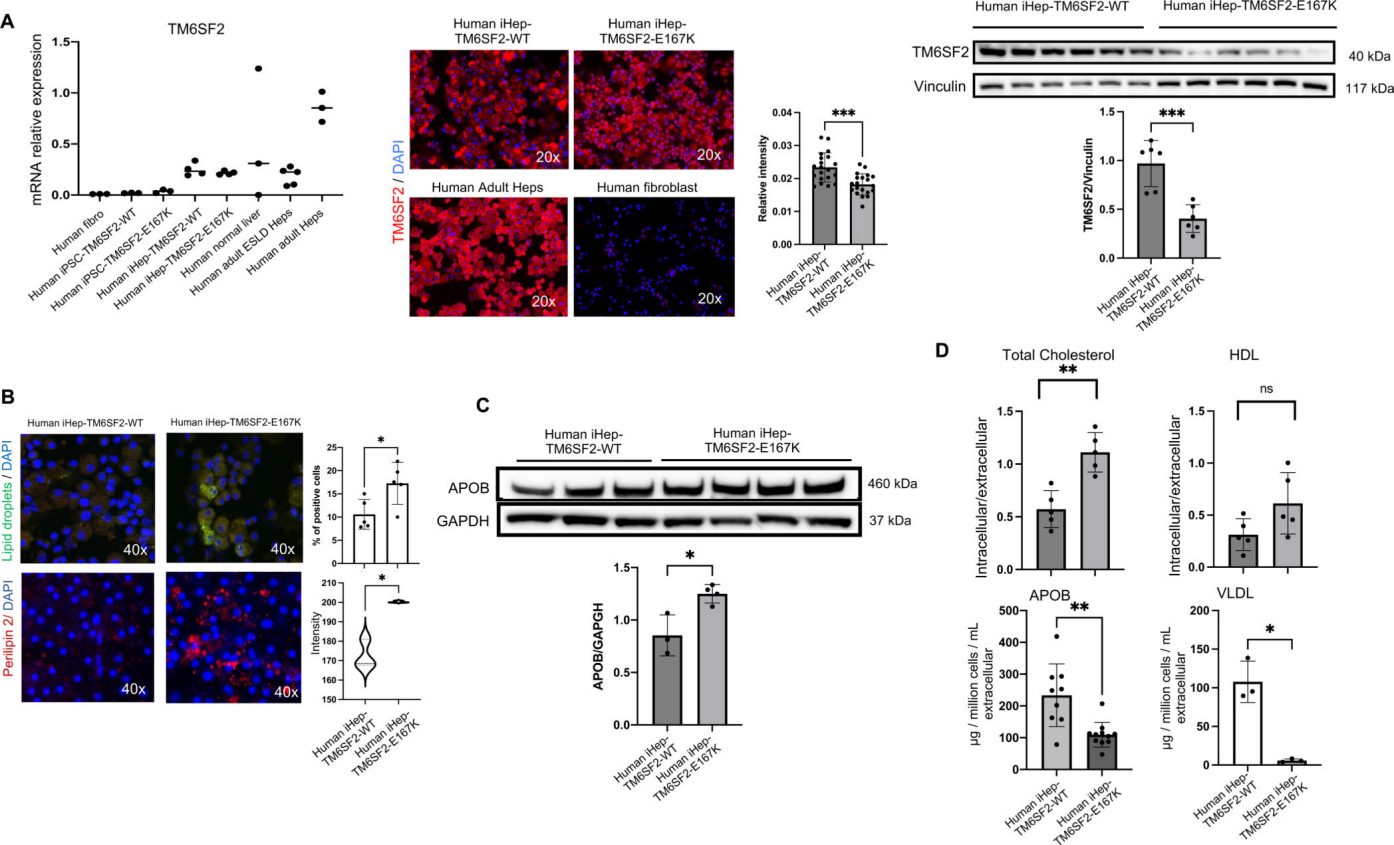
The TM6SF2-E167K mutation results in loss of function and alters lipid metabolism in human iHep. (A) *TM6SF2* expression in both iHep-TM6SF2-WT and iHep-TM6SF2-E167K. The left upper panel shows quantitative gene expression. iPSC-TM6SF2-WT (n = 3), iPSC-TM6SF2-E167K (n = 3), and human fibroblast cells (n = 3) were applied as negative controls. Human normal liver tissue (n = 3), human ESLD-WT hepatocytes (n = 5), and adult PHH (n = 3) were used as positive controls. Values are determined relative to β-actin and presented as fold change relative to the expression in PHH, which is set as 1. The middle upper panel shows immunofluorescence micrographs of the TM6SF2 marker in iHep-TM6SF2-WT and iHep-TM6SF2-E167K. Adult PHH was used as a positive control, and fibroblast as a negative control. The relative TM6SF2 intensity showed a significant decrease in iHep-TM6SF2-E167K cells when compared to iHep-TM6SF2-WT (mean ± SD ***p = 0.0002 unpaired Welch’s *t* test, n = 20 cells). The same was observed by western blot. The bar chart shows the quantification of protein expression. There was a significant decrease in iHep-TM6SF2-E167K in comparison to iHep-TM6SF2-WT (mean ± SD ***p = 0.0005, unpaired Welch’s *t* test, n = 6). (B) The upper panel shows Nile red staining micrographs in both iHep-TM6SF2-WT and iHep-TM6SF2-E167K and shows that iHep-TM6SF2-E167K has a higher intracellular lipid droplet content when compared to iHep-TM6SF2-WT. Quantification shows a significant increase in the percentage of Nile red signal when the cells carry the E167K mutation (mean ± SD *p = 0.0274, unpaired Welch’s *t* test n = 5). The lower panel shows Perilipin 2 staining micrographs in both iHep-TM6SF2-WT and iHep-TM6SF2-E167K and shows that iHep-TM6SF2-E167K has a higher intracellular lipid droplet content when compared to iHep-TM6SF2-WT. Quantification shows a significant increase in the intensity of the Perilipin 2 signal when the cells carry the E167K mutation (mean ± SD *p = 0.0229, unpaired Welch’s *t* test n = 3). (C) ApoB100 secretion is impaired in iHep-TM6SF2-E167K. The intracellular content of ApoB100 in iHep-TM6SF2-WT and iHep-TM6SF2-E167K was quantified by western blot. The bar chart shows an increase of ApoB100 inside the iHep-TM6SF2-E167K (mean ± SD *p = 0.0144, unpaired Welch’s *t* test). iHep-TM6SF2-WT (n = 3) and iHep-TM6SF2-E167K (n = 4). (D) Intracellular total cholesterol and HLD amounts were measured in iHep-TM6SF2-WT and iHep-TM6SF2-E167K. The bar charts show a significant increase in the intracellular and extracellular ratio of total cholesterol in iHep-TM6SF2-E167K when compared to iHep-TM6SF2-WT (mean ± SD **p = 0.0079, unpaired Welch’s *t* test, iHep-TM6SF2-WT, n = 4 and iHep-TM6SF2-E167K, n = 4). The secretion of ApoB100 in iHep-TM6SF2-WT and iHep-TM6SF2-E167K was evaluated by ELISA and showed a decrease of this apolipoprotein in iHep-TM6SF2-E167K (mean ± SD **p = 0.0042, unpaired Welch’s *t* test) in iHep-TM6SF2-WT (n = 9) and iHep-TM6SF2-E167K (n = 11). The secretion of VLDL in iHep-TM6SF2-WT and iHep-TM6SF2-E167K was evaluated by ELISA and showed a decrease of VLDL in iHep-TM6SF2-E167K (mean ± SD *p = 0.0217, unpaired Welch’s *t* test) in iHep-TM6SF2-WT (n = 3) and iHep-TM6SF2-E167K (n = 3).

Next, we studied the TM6SF2 transcript and protein expression. We found that TM6SF2 transcript levels were not significantly different between human iHeps-TM6SF2-WT, iHeps-TM6SF2-E167K, human liver tissue, or isolated human adult ESLD hepatocytes (Figure 3A). However, when protein expression was analyzed, iHeps-TM6SF2-E167K showed significantly reduced expression when compared to iHeps-TM6SF2-WT (Figure 3A), Supplemental Figure S2, http://links.lww.com/HEP/I675. A hallmark of MASLD progression, especially in individuals carrying the TM6SF2-E167K variant, is disruption of lipid and cholesterol regulation.^[10,22]^ Thus, we investigated the effect of the TM6SF2-E167K variant on intracellular and extracellular lipid accumulation by Nile red and Perilipin-2 staining. We observed a significant increase in the concentration of intracellular lipids in iHep-TM6SF2-E167K when compared to iHep-TM6SF2-WT (Figure 3B). Next, we determined the impact of the TM6SF2-E167K variant on cholesterol transporters, such as ApoB100 and VLDL, and found that intracellular ApoB100 protein expression and total cholesterol was significantly increased (Figure 3C) while extracellular secretion of ApoB100 and VLDL was significantly reduced in iHeps-TM6SF2-E167K (Figure 3D). We also measured levels of intracellular total cholesterol and other lipoprotein transporters, such as HDL, and found no significant differences.

### Global transcriptomic characterization of iHeps-TM6SF2-E167K revealed modified lipid metabolism

**FIGURE 4:**
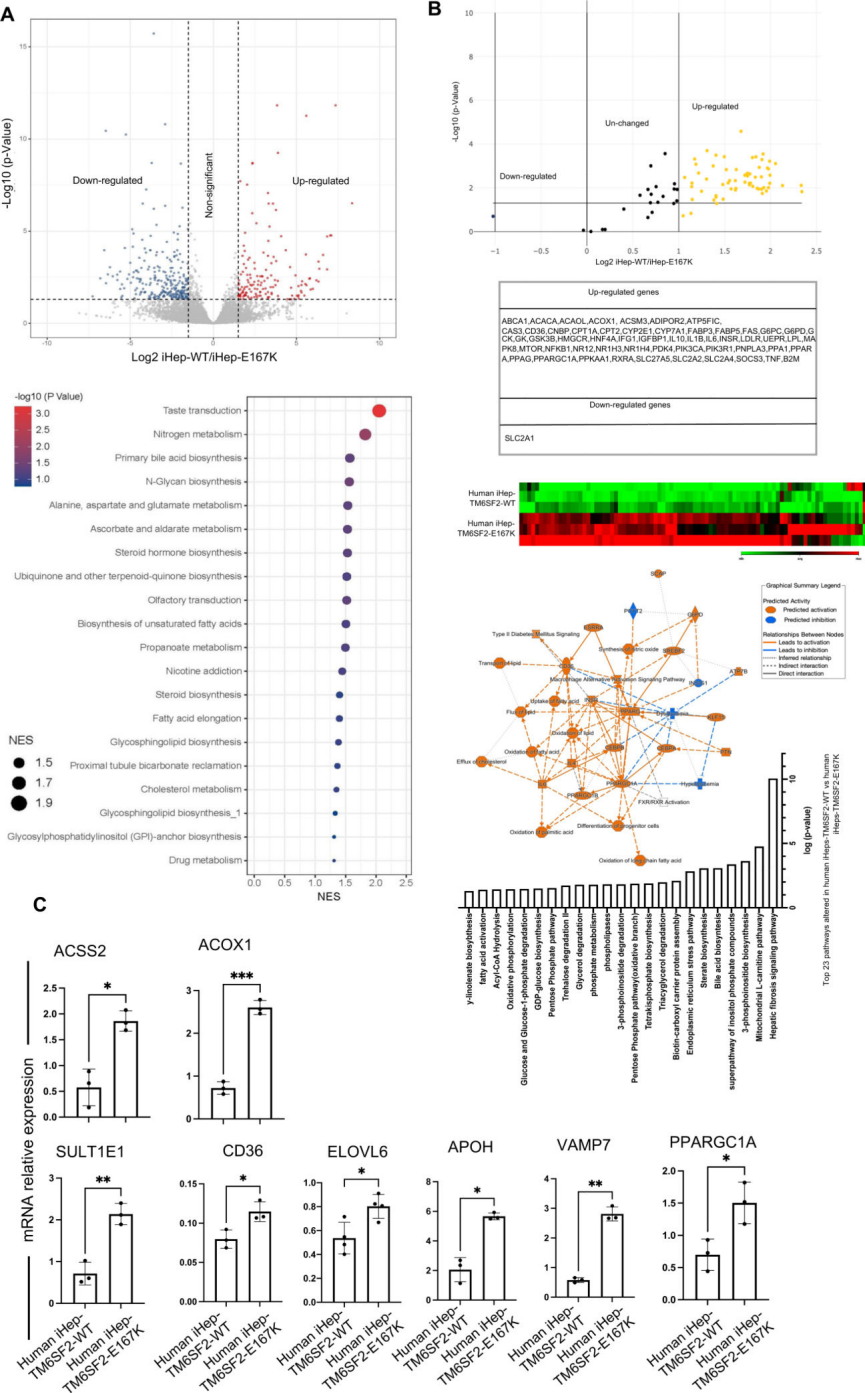
Transcription profiling analysis of iHep-TM6SF2-WT and iHep-TM6SF2-E167K. (A) Volcano plot showing the differential gene expression analysis of 3 independent differentiations of iHep-TM6SF2-WT and 4 independent differentiations of iHep-TM6SF2-E167K (left upper). The blue dots represent the downregulated genes, and the red dots represent the upregulated genes. The cutoff value for log_2_FC is 1.5 (adjust *p* = 0.05). The GSEA showed upregulated signaling pathways related to the TM6SF2-E167K mutation. (B) Volcano plot (upper panel) and heatmap (lower panel) showing human fatty liver metabolism RNA array analysis of 3 independent differentiations of iHep-TM6SF2-WT and iHep-TM6SF2-E167K. The table shows the upregulated and downregulated genes. Pathway analysis is based on the results from fatty liver metabolism RNA array analysis and the relationships between upstream regulators and biological functions. The top 23 pathways related to the iHep-TM6SF2-E167K mutation were ranked based on their *p* values. (C) Quantitative gene expression for important pathways highlighted by our differential expression and GSEA analysis. iHep-TM6SF2-E167K showed an increase in expression of ACSS2 (mean ± SD *p = 0.0109, unpaired Welch’s *t* test, n = 3), ACOX1 (mean ± SD ***p = 0.0001, unpaired Welch’s *t* test, n = 3), SULT1E1 (mean ± SD **p = 0.0027, unpaired Welch’s *t* test, n = 3), CD36 (mean ± SD *p = 0.0248, unpaired Welch’s *t* test, n = 3), ELOVL6 (mean ± SD *p = 0.0430, unpaired Welch’s *t* test, n = 4), APOH (mean ± SD *p = 0.0119, unpaired Welch’s *t* test, n = 3), VAMP7 (mean ± SD **p = 0.016, unpaired Welch’s *t* test, n = 3), and PPARGC1A (mean ± SD *p = 0.0290, unpaired Welch’s *t* test, n = 3) when compared to iHep-TM6SF2-WT. Values are determined relative to β-actin and presented as fold change relative to the expression in iHep-TM6SF2-WT, which is set as 1. Abbreviation: GSEA, gene set enrichment analysis.

Human livers undergo profound transcriptional and metabolic changes throughout the development of liver disease, and TM6SF2-E167K appears to influence disease progression.^[13]^ We analyzed the transcriptomic signature of TM6SF2-E167K using RNA-seq. Differential expression analysis revealed an upregulation of 153 genes and a downregulation of 267 genes (Figure 4A and Supplemental Table S4, http://links.lww.com/HEP/I636). Pathway enrichment analysis indicated an increase in the expression of genes related to cholesterol, fatty acid, and glucose metabolism in iHep-TM6SF2-E167K when compared to iHep-TM6SF2-WT. The upregulated pathways observed in gene set enrichment analysis are listed in Figure 4A.

To further corroborate the transcriptomic analysis, we performed a focused RNA array. Of the 56 upregulated genes, we observed an enrichment for genes associated with fatty acid oxidation (PPARGC1A, ACOX1, and ACSM3), uptake of fatty acids (CD36), lipid transportation (CPT1A and CPT2), and glucose metabolism (SLC2A2 and SLC2A4) in iHep-TM6SF2-E167K (Figure 4B). Further pathway analyses unveiled elevated activities related to the transport, flow, removal, and oxidation of lipids, fatty acids, and cholesterol (Figure 4B). We confirmed our RNA-seq findings using qPCR, focusing specifically on the most important pathways highlighted by our differential expression and gene set enrichment analysis (fatty acid oxidation: ACOX1 and PPARGC1A; fatty uptake: CD36; lipid synthesis and transportation: ACSS2 and VAMP7; steroid hormone biosynthesis: SULT1E1; fatty acid elongation: ELOVL6; cholesterol metabolism: APOH; Figure 4C). For all genes, we observed a significant increase in iHep-TM6SF2-E167K when compared to iHep-TM6SF2-WT, confirming our RNA-seq results. We also performed a comparison to transcriptomic data from population studies and confirmed at least 5 common pathways related to cholesterol metabolism, synthesis and metabolism of fatty acids, and synthesis of steroids (Supplemental Figure S3, http://links.lww.com/HEP/I637).

As we observed an upregulation of gene expression related to glucose metabolism in our fatty liver metabolism RNA array, we evaluated insulin resistance in iHep-TM6SF2-E167K. The signaling pathway leading to AKT activation is disrupted when insulin resistance is present in hepatocytes, resulting in decreased phospho-AKT. Phospho-AKT is crucial in mediating the metabolic actions of insulin.^[25]^ Although there was an upregulation in glucose metabolism, no significant change was observed in phospho-AKT protein levels in our iHep-TM6SF2-E167K, suggesting no insulin resistance iHep-TM6SF2-E167K when compared to iHep-TM6SF2-WT (Supplemental Figure S4, http://links.lww.com/HEP/I638).

### TM6SF2-E167K variant modifies lipid metabolism in human iHeps

**FIGURE 5:**
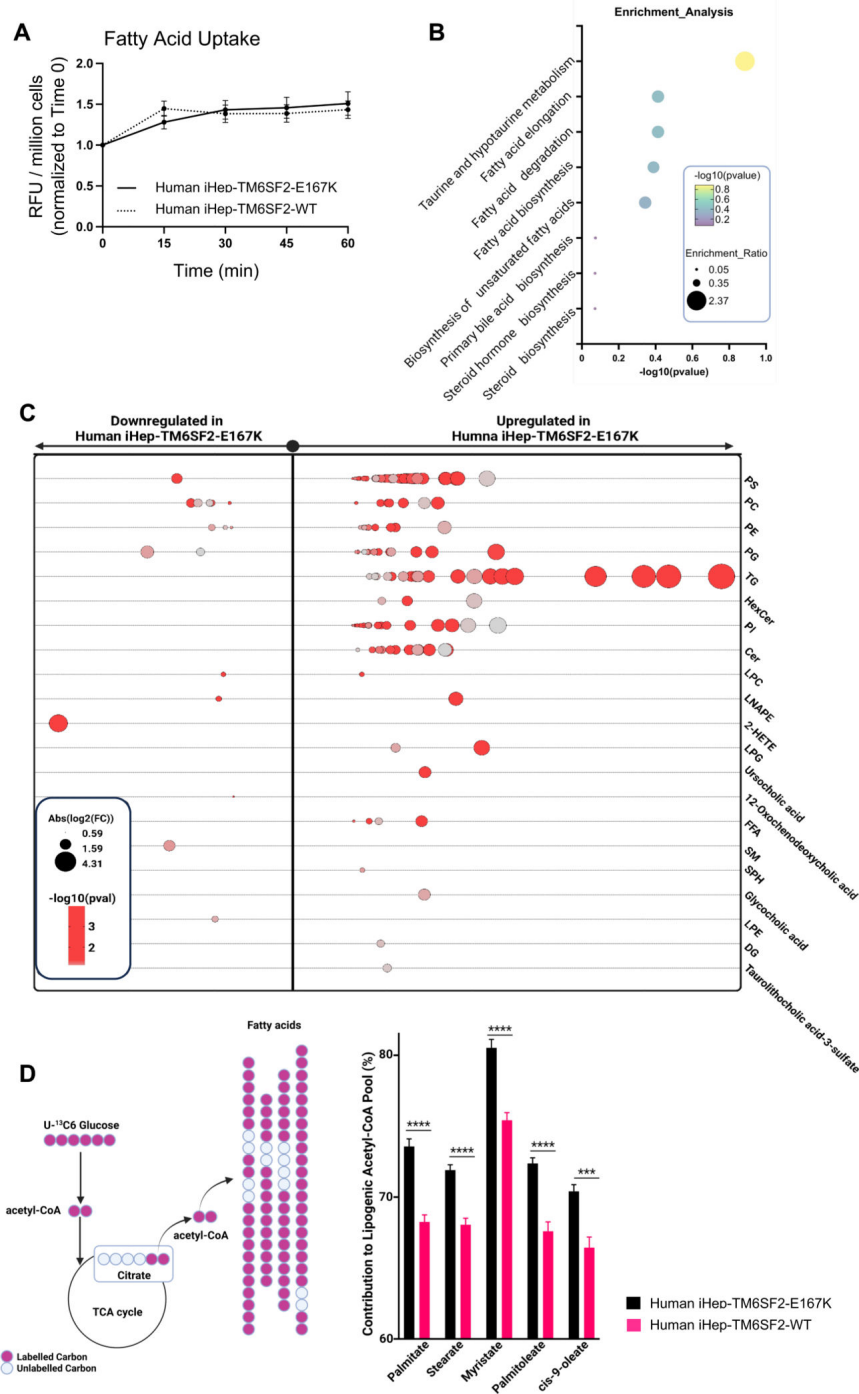
The TM6SF2-E167K variant modifies lipid metabolism in human iHeps. (A) Kinetics of fatty acid uptake in iHep-TM6SF2-WT and iHep-TM6SF2-E167K. Fatty acid uptake was measured using a fluorescent fatty acid analog, and the data are represented as RFU/millions of cells and normalized to time zero (n = 3). The uptake of fatty acids was assessed at 0,15, 30, 45, and 60 minutes. No differences were observed in iHep-TM6SF2-WT when compared to iHep-TM6SF2-E167K. (B) Pathway enrichment analysis on global lipidomics data. The analysis indicates an over-representation of lipids in the different pathways of interest. The data show an increased activity in the fatty acid synthesis pathway in mutant cells, which is one of the major building blocks for other lipids. The analysis was done with Metaboanalyzt (version 6.0). (C) Bubble plot showing the fold changes of various intracellular lipids of different classes in human iHep-TM6SF2-E167K (n = 3) when compared to the iHep-TM6SF2-WT (n = 3). The majority of the lipids measured in various classes have significant upregulation in the iHep-TM6SF2-E167K group when compared to the iHep-TM6SF2-WT group. The size of the circles represents the Log2 (fold change). (D) ISA shows significant utilization of glucose toward increasing the cytosolic acetyl-CoA in iHep-TM6SF2-E167K (n = 3) when compared to iHep-TM6SF2-WT (n = 3). Test statistics were calculated based on unpaired *t* test with Welch correction using Graphpad prism. Palmitate 16:0 (****p < 10–4), Stearate 18:0 (****p < 10–4), Myristate 14:0 (****p < 10–4), Palmitoleate 16:1 (****p < 10–4) cis-9-oleate 18:1 (***p = 0.000187). Inserted is a schematic depicting the mechanism by which glucose contributes labeled carbon into acetyl-CoA. Abbreviations: Cer, Ceramides; DG, diacylglycerols; FFA, free fatty acid; 2-HETE, hydroxyeicosatetraenoic acids; HexCer, hexosylceramide; ISA, isotopolog spectral analysis; LNAPE, *N*-acyl-lysophosphatidylethanolamines; LPC, lysophosphatidylcholines; LPE, lysophosphatidylethanolamine; LPG, Lyso-phosphatidylglycerol; PC, phosphatidylcholine; PE, phosphatidyethanolamine; PI, phosphatidylinositol; PS, phosphatidylserine; SM, sphingomyelin; SPH, sphingosine; TG, triglyceride.

To understand what is leading to lipid accumulation in iHep-TM6SF2-E167K, we investigated lipid synthesis, uptake, and degradation. To evaluate fatty acid uptake, we used a fluorescent long-chain fatty acid analog and determined the uptake kinetics in live cells. We did not observe any difference in fatty acid uptake (Figure 5A) when comparing both groups. To assess the effect of the TM6SF2 mutation on lipid metabolism, we performed a detailed global lipidomic analysis. Pathway enrichment analysis^[26]^ indicated an over-representation of lipids in pathways related to fatty acid synthesis and degradation (Figure 5B). Moreover, iHep-TM6SF2-E167K showed significant upregulation of the majority of intracellular lipid classes, including triglycerides (TG), phospholipids (PS, PC, PE, PI, and LPC), bile acids (tauroluthocholic acid, glycocholic acid, and 12-Oxochenodeoxycholic acid), glycerol lipids (DG), free fatty acids, lysophospholipids (LPE, LNAPE, and LPG), and sphingolipids (SM, SPH, and CER)^[27]^ (Figure 5C) when compared to iHep-TM6SF2-WT. These types of lipids are normally observed in the lipid droplet core (neutral lipids including TGs) and the outer phospholipid monolayer (PC and PS, among others).^[28]^

Fatty acid synthesis is a complex and highly regulated process essential for the production of fatty acids, which serve as building blocks for various lipids and play critical roles in energy metabolism and cellular function.^[29]^ We performed stable isotope tracing experiments using uniformly labeled glucose as the substrate and measured the isotopologs of various medium and long-chain fatty acids, including palmitate, stearate, myristate, palmitoleate, and cis-9-oleate. Our results showed a significant increase in the fraction of high-mass isotopologs derived from glucose (Supplemental Figure S5, http://links.lww.com/HEP/I639) for all of the aforementioned fatty acids in iHep-TM6SF2-E167K. In addition, we performed isotopolog spectral analysis^[30]^ to estimate the contribution of glucose to cytosolic acetyl-CoA. The major building block for fatty acid synthesis is acetyl-CoA. One of the major sources of acetyl-CoA is glycolysis, in which glucose is broken into pyruvate, enters the TCA cycle, and generates acetyl-CoA. This acetyl-CoA forms long-chain fatty acids that are incorporated into triacylglycerol, phospholipids, and cholesterol esters in hepatocytes, which are stored in lipid droplets. Our results from the tracing experiments suggest that there is a significantly higher production of acetyl-CoA contributing to fatty acid synthesis in iHep-TM6SF2-E167K cells when compared to iHep-TM6SF2-WT (Figure 5D). Taken together, the glucose tracing and global lipidomics analysis significantly support our hypothesis that E167K mutation alters lipid synthesis.

### Characterization of iHeps-TM6SF2-E167K revealed cellular stress

**FIGURE 6:**
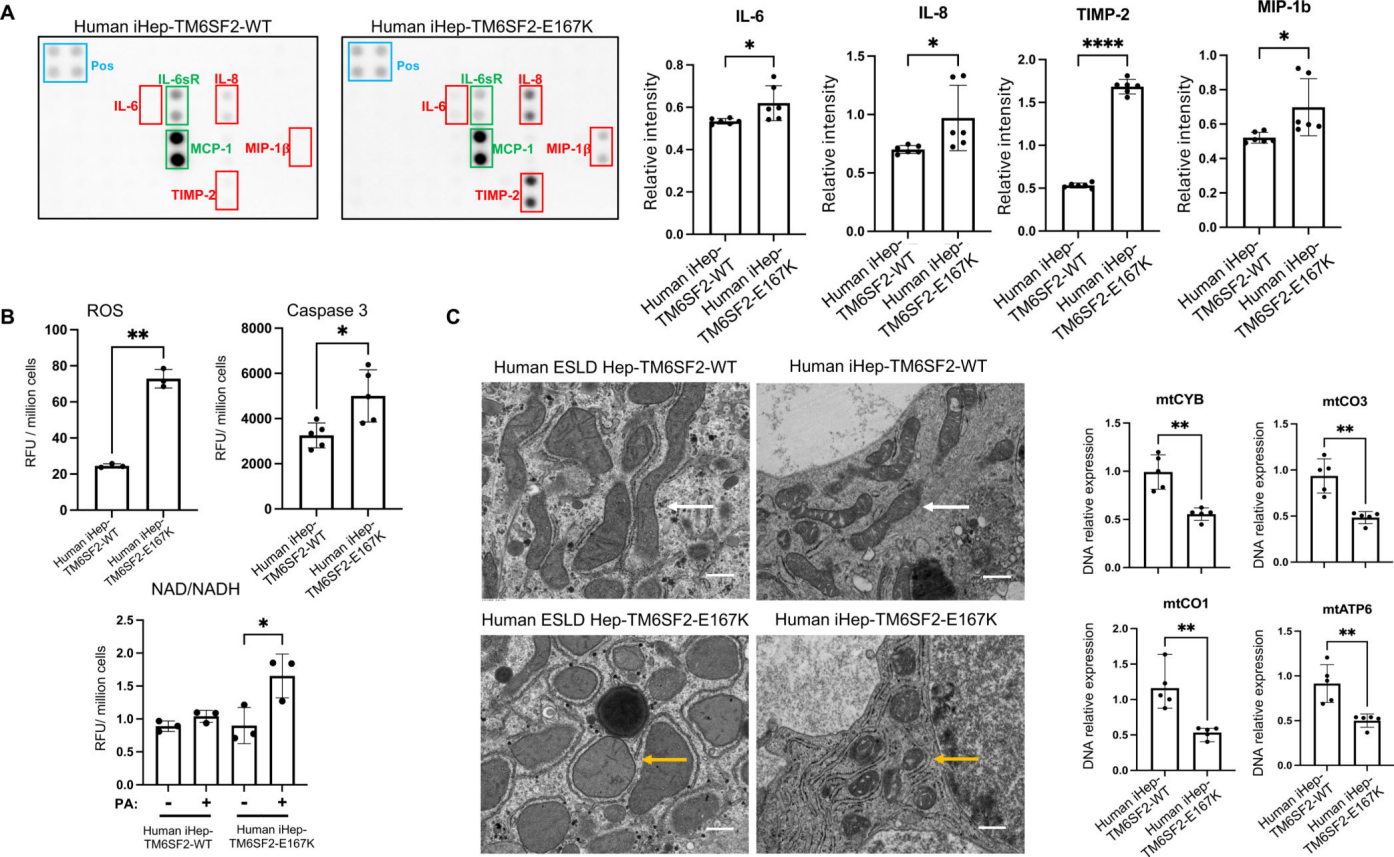
Characterization of iHeps-TM6SF2-E167K revealed cellular stress. (A) The inflammatory response in iHep-TM6SF2-WT and iHep-TM6SF2-E167K was quantified by Multiplex Protein Detection. The representative images of human inflammatory array membranes show the expression analysis of 3 independent differentiations of iHep-TM6SF2-WT and 3 independent differentiations of iHep-TM6SF2-E167K. The blue rectangle represents the experimental positive control, the red rectangles represent downregulated cytokines and chemokines in iHep-TM6SF2-E167K, and the green rectangles represent upregulated cytokines and chemokines in iHep-TM6SF2-WT. After the development of the membranes, images were scanned and analyzed using ImageJ software. All dot density values were normalized to the dot density for positive control. The bar charts show an increase of IL-6 (mean ± SD *p = 0.0307, unpaired Welch’s *t* test), IL-8 (mean ± SD *p = 0.0410, unpaired Welch’s *t* test), MIP-1β (mean ± SD *p = 0.0279, unpaired Welch’s *t* test), and TIMP-2 (mean ± SD ****p < 0.0001, unpaired Welch’s *t* test), levels in iHep-TM6SF2-E167K when compared to iHep-TM6SF2-WT. (B) The bar chart shows that total ROS is significantly increased in iHep-TM6SF2-E167K when compared to iHep-TM6SF2-WT (mean ± SD **p = 0.0026, unpaired Welch’s *t* test n = 3). Total Caspase 3 measurement shows a significant increase in iHep-TM6SF2-E167K when compared to iHep-TM6SF2-WT (mean ± SD *p = 0.0317, unpaired Welch’s *t* test, n = 5). Total NAD/NADH quantification in iHep-TM6SF2-WT and iHep-TM6SF2–167K treated with 100 μM of PA for 48 hours shows a significant increase of NAD/NADH in iHep-TM6SF2-E167K treated when compared to iHep-TM6SF2-E167K nontreated (mean ± SD *p = 0.0407, unpaired Welch’s *t* test, n = 3). No difference was observed in iHep-TM6SF2-WT treated and nontreated (mean ± SD *p* = 0.0975, unpaired Welch’s *t* test, n = 3). (C) TEM images of ESLD-TM6SF2-WT (n = 1), ESLD-TM6SF2-E167K (n = 1), iHep-TM6SF2-WT (n = 3), and iHep-TM6SF2-E167K (n = 3). The white arrows indicate the rod mitochondrial shape commonly found in human hepatocytes. The yellow arrows show the round mitochondrial shape in hepatocytes that carried the TM6SF2-E167K mutation (scale bar: 600 nm). The right panel shows DNA quantitative gene expression for important functional mitochondria genes. iHep-TM6SF2-E167K showed a decrease in expression of mtCYB (mean ± SD **p = 0.0079, unpaired Welch’s *t* test, n = 5), mtCO3 (mean ± SD **p = 0.0079, unpaired Welch’s *t* test, n = 5), mtCO1 (mean ± SD **p = 0.0079, unpaired Welch’s *t* test, n = 5), and mtATP6 (mean ± SD **p = 0.0079, unpaired Welch’s *t* test, n = 5) when compared to iHep-TM6SF2-WT. Values are determined relative to β-actin and presented as fold change relative to the expression in iHep-TM6SF2-WT, which is set as 1. Abbreviation: TEM, transmission electron microscopy.

Excessive accumulation of lipids in hepatocytes can lead to ER stress,^[31]^ as the ER is unable to properly process and fold proteins or metabolize lipids,^[32]^ and the activation of inflammatory signaling pathways.^[33]^ By performing a multiplex protein detection approach, we observed increases in proinflammatory cytokines (IL-6 and IL-8) and chemokines (MIP-1β) along with elevated TIMP-2 (indicator of fibrogenesis) in iHep-TM6SF2-E167K (Figure 6A). This suggests a pivotal role of the TM6SF2-E167K mutation in promoting hepatic inflammation and fibrogenesis. As cellular stress may result in ROS production,^[33]^ we evaluated ROS accumulation and observed a significant increase in iHep-TM6SF2-E167K when compared to controls (Figure 6B). In addition, intracellular cholesterol accumulation can lead to an increase in beta-oxidation.^[34]^ We confirmed increased NADH, a product of beta-oxidation, in iHep-TM6SF2-E167K (Figure 6B). To understand if E167K increased hepatocyte death, we measured Caspase-3 levels and found a significant increase in Caspase-3 levels in iHep-TM6SF2-E167K cells (Figure 6B). This observation suggests a potential enhancement in apoptotic activity associated with the TM6SF2-E167K variant.

Finally, we found an increased number of spherical mitochondria (Figure 6C; yellow arrow) among human hepatocytes ESLD-E167K and iHep-TM6SF2-E167K when compared to control human hepatocytes ESLD-WT and iHep-TM6SF2-WT. Maintenance of the mitochondrial genome is essential for proper cellular function; thus, we analyzed the expression of important mitochondrial genes: mitochondrial cytochrome b (mtCYB), mitochondrial cytochrome c oxidase subunit I (mtCO1), mitochondrial cytochrome c oxidase subunit III (mtCO3), and mitochondrial ATP synthase subunit 6 (mtATF6). These genes are found in the mitochondrial DNA and ensure proper energy production (mtATF6), regulation of metabolic pathways (mtCO1 and mtCYB), and maintenance of mitochondrial integrity (mtCO3).^[35]^ As expected, these genes were significantly lower in iHEP-TM6SF2-E167K when compared to iHep-TM6SF2-WT (Figure 6C).

### TM6SF2-E167K confers ER stress to iHeps that is alleviated by facilitating protein folding

**FIGURE 7:**
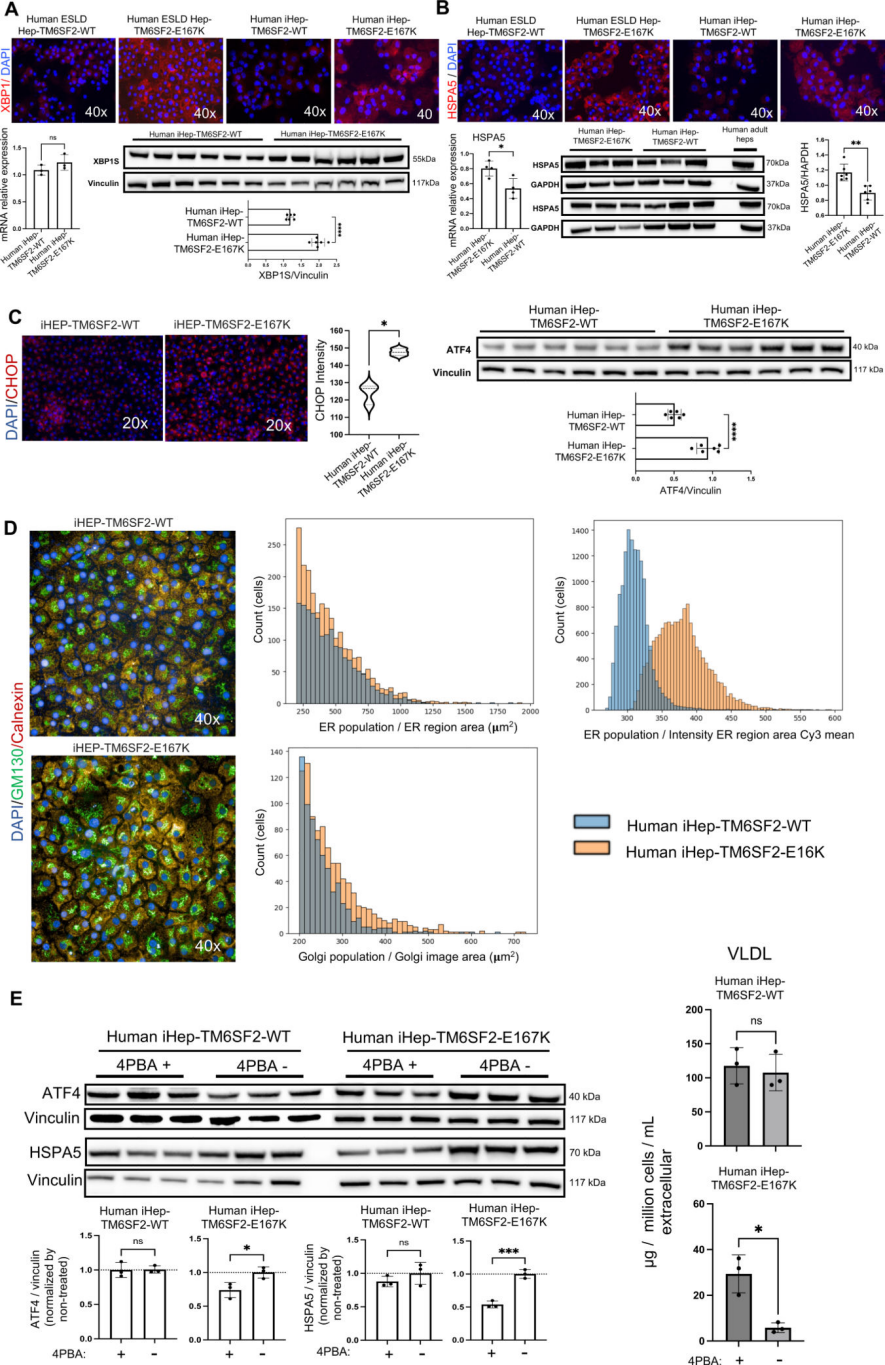
TM6SF2-E167K mutation is related to ER stress. (A) Immunofluorescence micrographs of XBP1, a protein related to ER stress, in ESLD-TM6SF2-WT (n = 1), ESLD-TM6SF2-E167K (n = 1), iHep-TM6SF2-WT (n = 3), and iHep-TM6SF2-E167K (n = 3), showing an increase in protein expression in the mutant samples (ESLD-TM6SF2-E167K and iHep-TM6SF2-E167K). The relative XBP1 expression was not significantly different in iHep-TM6SF2-E167K when compared to iHep-TM6SF2-WT (mean ± SD *p* = 0.4000, unpaired Welch’s *t* test n = 3). The opposite was observed by western blot. The bar chart shows the quantification of XBP1s protein expression, and a significant increase was observed in iHep-TM6SF2-E167K in comparison to iHep-TM6SF2-WT (mean ± SD ****p < 0.0001, unpaired Welch’s *t* test n = 6). (B) Immunofluorescence micrographs of HSPA5, a protein related to ER stress, in ESLD-TM6SF2-WT (n = 1), ESLD-TM6SF2-E167K (n = 1), iHep-TM6SF2-WT (n = 3), and iHep-TM6SF2-E167K (n = 3), showing an increase in protein expression in the mutant samples (ESLD-TM6SF2-E167K and iHep-TM6SF2-E167K). The relative HSPA5 expression showed a significant increase in iHep-TM6SF2-E167K when compared to iHep-TM6SF2-WT (mean ± SD *p = 0.0188, unpaired Welch’s *t* test n = 4). Values are determined relative to β-actin. The same was observed by western blot. The bar chart shows the quantification of HSPA5 protein expression, and a significant increase was observed in iHep-TM6SF2-E167K in comparison to iHep-TM6SF2-WT (mean ± SD **p = 0.0010, unpaired Welch’s *t* test n = 6). (C) Immunofluorescence micrographs of CHOP, a protein related to ER stress, in iHep-TM6SF2-WT (n = 3) and iHep-TM6SF2-E167K (n = 3), showing an increase in protein expression in iHep-TM6SF2-E167K. The bar chart shows that the intensity quantification of CHOP is significantly increased in iHep-TM6SF2-E167K in comparison to iHep-TM6SF2-WT (mean ± SD *p = 0.0118, unpaired Welch’s *t* test n = 3). The same was observed by western blot for AFT4, another protein related to ER stress. The bar chart shows that ATF4 protein expression is significantly increased in iHep-TM6SF2-E167K in comparison to iHep-TM6SF2-WT (mean ± SD ****p < 0.0001, unpaired Welch’s *t* test n = 6). (D) ER and Golgi expression in both iHep-TM6SF2-WT and iHep-TM6SF2-E167K. Immunofluorescence micrographs show calnexin marking ER in red and GM130 marking Golgi in green (×40, n = 3). The histograms show that there is no difference in ER region area in iHep-TM6SF2-E167K when compared to iHep-TM6SF2-WT. The opposite was observed in the Golgi area and ER intensity, where the histograms show a significant difference in iHep-TM6SF2-E167K when compared to iHep-TM6SF2-WT. (E) After treatment with 2 μM of 4PBA for 48 hours, ATF4 and HSPA5 protein expression was observed by western blot in both iHep-TM6SF2-WT and iHep-TM6SF2-E167K. The bar chart shows the quantification of ATF4 and HSPA5 normalized to nontreated cells. ATF4 and HSPA5 showed a significant increase in expression in iHep-TM6SF2-E167K treated when compared to nontreated iHep-TM6SF2-E167K (ATF4: mean ± SD *p = 0.0313, unpaired Welch’s *t* test n = 3), (HSPA5: mean ± SD ***p = 0.0007, unpaired Welch’s *t* test n = 3). No difference in either protein was observed in iHep-TM6SF2-WT treated when compared to nontreated iHep-TM6SF2-WT. The quantification of VLDL secretion showed no difference in iHep-TM6SF2-WT treated when compared to nontreated iHep-TM6SF2-WT (mean ± SD *p* = 0.6752, unpaired Welch’s *t* test n = 3) and a significant increase in the secretion of VLDL in iHep-TM6SF2-E167K treated when compared to nontreated iHep-TM6SF2-E167K (mean ± SD *p = 0.0321, unpaired Welch’s *t* test n = 3).

We analyzed genes indicative of ER and mitochondrial stress, such as X-box binding protein 1 (XBP1), a transcription factor that plays a key role in ER stress,^[36]^ and heat shock protein family A (Hsp70) member 5 (HSPA5), a chaperone protein in the ER that helps fold and assemble proteins and is highly expressed during stress.^[37]^ We found that both XBP1 and HSPA5 proteins, as assessed by immunofluorescence, were increased in iHep-TM6SF2-E167K to levels observed in human hepatocytes freshly isolated from livers with ESLD and carrying the TM6SF2-E167K variant (Figures 7A, B). This observation was corroborated by the western blot for XBP1s (Figure 7A), but qPCR did not show any difference between both groups. For HSPA5, the increase in iHep-TM6SF2-E167K was also observed by qPCR and western blot (Figure 7B). To further support our findings, we evaluated other ER stress markers, CHOP and ATF4, both of which were increased in iHep-TM6SF2-E167K when compared with iHep-TM6SF2-WT (Figure 7C). We did not observe any differences in ER location, but a significant increase in Golgi area and ER fluorescence intensity was observed in iHep-TM6SF2-E167K (Figure 7D). Taken together, these data demonstrate that alterations in lipid synthesis driven by the TM6SF2-E167K variant can increase ER stress.

The ER is essential for the folding and trafficking of proteins that enter the secretory pathway. The main characteristic of ER stress is often protein misfolding, leading to cell death. Thus, we reasoned that alleviating protein misfolding induced by the TM6SF2-E167K variant could improve cell function. 4PBA is an aromatic fatty acid that has been investigated for improving protein misfolding and ER stress.^[38]^ Studies have shown that TM6SF2-E167K mutation results in a misfolded protein, accelerated protein degradation, and reduced protein levels, contributing to the observed phenotypes.^[10,39]^ After treating iHep-TM6SF2-E167K cells with this compound, we observed a significant decrease in ATF4 and HSPA5 protein levels compared to untreated cells. To understand if this reduction influenced lipid secretion, we tested VLDL levels and observed a significant increase in VLDL secretion in treated iHep-TM6SF2-E167K compared to untreated cells. No differences in ATF4, HSPA5, or VLDL secretion were observed between treated and untreated iHep-TM6SF2-WT (Figure 7E). This suggests that treating protein misfolding induced by TM6SF2-E167K can lead to reductions in ER stress and lipid accumulation.

## DISCUSSION

There is increasing evidence that *TM6SF2* plays a significant role in the metabolic processing of hepatic lipids. Lipotoxicity within the liver can trigger inflammation, oxidative stress, and cellular injury, ultimately contributing to the development of MASLD.^[40,41]^ MASLD is marked by an abundance of fat accumulating in the liver^[42]^ and encompasses a spectrum of conditions, ranging from simple fat accumulation (steatosis) to more severe disorders like MASH.^[43]^ As this allele is relatively rare in primary tissue, researchers have invested in animal studies. However, these mice do not accurately reflect the consequences of genetic mutation. Moreover, the mouse and human^[44]^ TM6SF2 proteins are only 78% identical. We generated iHep-TM6SF2-E167K to create an accurate model to study the impact of *TM6SF2* rs58542926 on hepatocyte function. In this study, we generated iPSCs from a healthy individual, followed by CRISPR/Cas9 gene editing to introduce the variant.^[23]^ Induced hepatocytes demonstrated upregulation in lipid accumulation, total cholesterol, intracellular ApoB100, ER and mitochondria stress markers, ROS, beta-oxidation, apoptosis markers, proinflammatory molecules, and fatty acid biosynthesis pathways.

The negative effect of intracellular lipid accumulation has been previously described.^[45,46]^ We observed an increase in lipid droplets inside iHep-TM6SF2-E167K when compared to iHep-TM6SF2-WT. Furthermore, we analyzed both intracellular and extracellular ApoB100, VLDL, and cholesterol levels. VLDL transports triglycerides from the liver to peripheral tissues, while ApoB100 is essential for the formation, stability, and function of VLDL particles.^[47]^ Dysregulation of VLDL and ApoB100 production and metabolism can lead to lipid disorders and contribute to conditions like MASLD.^[48]^ Our studies revealed a significant difference in total cholesterol, with a notable variation in the ratio between intracellular and extracellular content. A significant decrease in VLDL and ApoB100 secretion in iHep-TM6SF2-E167K accompanied by higher intracellular ApoB100 was observed. In 2019, Prill et al^[22]^ demonstrated that having only one allele copy (CT) of the TM6SF2-E167K mutation is linked to an upregulation of cholesterol and fatty acid biosynthesis pathways, along with decreased ApoB100 secretion in 3D spheroid cultures of primary human mutant hepatocytes. Divergent results were found in animal models in which the quantity of ApoB100 particles secreted by *TM6SF2* KO mice remained unchanged.^[18]^ These conflicting findings raise questions about the extent to which results from mouse models can be extrapolated to human physiology, emphasizing the need for clinical metabolic research.^[49,50]^ Our results indicate overall cellular damage, corroborated by the upregulation of genes involved in cholesterol, fatty acid, and glucose metabolism in iHep-TM6SF2-E167K.

We also noted heightened expression of genes and proteins associated with mitochondrial dysfunction and ER stress in iHep-TM6SF2-E167K, indicating potential overall cellular damage.^[51,52]^ It is well established that HSP70, a crucial protein associated with mitochondrial and ER stress, plays a vital role in the generation, proper folding, and transportation of misfolded proteins to proteolytic enzymes within the mitochondrial matrix. Moreover, we detected irregularities in mitochondrial structure, as evidenced by transmission electron microscopy, aligning with known characteristics of ER stress.^[53]^ Global lipidomics analysis showed an upregulation of important lipid classes that are related to ER stress,^[54]^ mitochondrial dysfunction,^[55]^ apoptosis,^[56]^ absorption and excretion of cholesterol, lipid metabolism, and the breakdown of triglycerides.^[57,58]^ Earlier studies have established that TM6SF2-E167K leads to protein misfolding, acceleration of protein degradation, and a reduction in TM6SF2 protein levels and function.^[59,60]^ To elucidate the mechanistic link between the TM6SF2-E167K variant, cellular stress, and lipid metabolism, we treated iHep-TM6SF2-E167K with an aromatic fatty acid, 4BBA, that has potential therapeutic effects against protein misfolding and ER stress.^[38,61]^ After facilitating protein folding therapy, we observed a significant decrease in ATF4 and HSPA5 levels, 2 major branches of the unfolded protein response.^[62]^ Protein folding facilitation therapy can impact lipid metabolism, as ER stress influences lipid synthesis and storage.^[63]^ By alleviating the negative effects of UPR, 4PBA can help restore ER lipid homeostasis by downregulating SREBP1c, a crucial transcription factor that regulates the expression of genes involved in lipid metabolism, such as ACC and FASN.^[63–65]^ This downregulation reduces lipid accumulation, mitigating lipotoxicity-induced ER stress, and helps maintain a balanced lipid profile. In addition, by reducing ER stress, 4PBA can decrease inflammatory phenotype, which is often linked to metabolic diseases and can worsen lipid metabolism disorders.^[66]^ The observed decrease in ER stress markers in response to 4PBA treatment in iHep-TM6SF2-E167K highlights the therapeutic potential of targeting protein misfolding to alleviate ER stress and mitigate the detrimental effects of the TM6SF2-E167K mutation on hepatic lipid metabolism. These findings highlight one of the mechanisms by which this TM6SF2 variant increases susceptibility to liver disorders.

The biggest limitation of our study is the small number of human hepatocytes derived from individuals carrying the TM6SF2-E167K to use as controls due to the relatively low frequency of the E167K variant. As a result, we were unable to investigate the expression pattern of the E167K variant in other hepatic cell types in normal liver tissue. Although no difference was observed in the number of positive cells for TM6SF2 in human livers with ESLD carrying either the TM6SF2-WT or the TM6SF2-E167K polymorphism (data not shown), we could not assess the TM6SF2 expression pattern and functional interactions on nonparenchymal cells. However, the human iPSC-based model system developed here will be useful for further investigation of the role of *TM6SF2* rs58542926 in the evaluation of functional differences in nonparenchymal cells (endothelial, stellate, and KCs) and future experiments will be focused to determine the degree to which they contribute to the observed effects, helping in the identification of the mechanism by which this variant increases the risk for liver disease in patients.

## Supplementary Material

Sup Fig 3

Sup Figure 1

Sup Fig 2

Sup Fig 4

Sup Tab 1

Sup Fig 5

Sup Tab 2

Sup Tab 3

Sup Tab 4

Sup Tab 5

## Abbreviations:

4PBA: 4-Phenylbutyric acid
ACC: acetyl-CoA carboxylase
ApoB: apolipoprotein B
CLD: chronic liver disease
ER: endoplasmic reticulum
FASN: fatty acid synthase
GCKR: glucokinase regulator
iPSC: human-induced pluripotent stem cell
iHeps: induced hepatocytes
MBOAT7: membrane bound *O*-acyltransferase domain-containing 7
MASH: metabolic dysfunction–associated steatohepatitis
MASLD: metabolic dysfunction–associated steatotic liver disease
mtATF6: mitochondrial ATP synthase subunit 6
mtCYB: mitochondrial cytochrome b
mtCO1: mitochondrial cytochrome c oxidase subunit I
mtCO3: mitochondrial cytochrome c oxidase subunit III
PNPLA3: patatin-like phospholipase domain-containing protein 3
SREBP1c: sterol regulatory element-binding transcription factor 1
TM6SF2: transmembrane 6 superfamily 2
XBP1: X-box binding protein 1
