# Supplementary material for: Human-induced pluripotent stem cell–based hepatic modeling of lipid metabolism–associated TM6SF2-E167K variant
Source: Hepatology. 2024 Aug 27;82(3):638–54. doi: 10.1097/HEP.0000000000001065 (PMC11865362; doi:10.1097/HEP.0000000000001065)
Supplement: Supplementary file 1 [file hep-82-0638-s001.docx]

**Supplementary table 1: Characteristics of human cells used in this study.**
