# Supplementary material for: Human-induced pluripotent stem cell–based hepatic modeling of lipid metabolism–associated TM6SF2-E167K variant
Source: Hepatology. 2024 Aug 27;82(3):638–54. doi: 10.1097/HEP.0000000000001065 (PMC11865362; doi:10.1097/HEP.0000000000001065)
Supplement: Supplementary file 6 [file hep-82-0638-s006.docx]

**Methods**

**Primary Human Hepatocytes**

Cryopreserved primary hepatocytes from healthy individuals were obtained from In Vitro ADMET Laboratories Inc. (IVAL, Columbia, MD, USA). End-stage liver disease (ESLD) hepatocytes were isolated from therapeutically resected livers and fresh human liver tissue specimens from patients (IRB: STUDY20090069) undergoing liver transplantation in the adult liver transplant programs at the University of Pittsburgh Medical Center (UPMC). Human fetal liver tissues were obtained from the University of Washington Department of Pediatrics, Division of Genetic Medicine, Laboratory of Developmental Biology (Seattle, WA) after obtaining written informed consent by a protocol approved by the Human Research Review Committee of the University of Pittsburgh (honest broker approval numbers HB015 and HB000836). Human fetal liver hepatocytes were isolated, cultured, and differentiated into fibroblasts, as previously described (1). Specific information on the age, gender, and cell viability of human liver tissue and hepatocytes used in this study is described in Supplementary Table 1 and Supplementary Table 5.

**Generation and Culture of Human iPSC**

iPSC-TM6SF2-WT was generated from fibroblasts. Fibroblasts were reprogrammed using episomal plasmid vectors adapted from a previously described method (21). Briefly, for each nucleofection, 1 million cells were resuspended in 100 mL of the AmaxaTM NHDF Nucleofector Kit (Lonza, Walkersville, MD), containing 1 μg of each of the four episomal plasmid vectors encoding OCT3/4 and p53 shRNA, SOX2 and KLF4, L-MYC and LIN28, and enhanced green fluorescent protein (eGFP) (Addgene, Boston, MA). Cells were nucleofected using the Amaxa 4D-Nucleofector (Lonza, Walkersville, MD) and plated in mTeSR on human embryonic stem cell-qualified Matrigel-coated plates (Corning, New York, NY). Colonies were isolated around 60 days after induction based on morphology. These cell lines underwent karyotyping, and pluripotency was validated by the expression of NANOG, OCT4, and membrane markers SSEA and TRA-1-60 at different passages. Additionally, the cell lines were routinely tested and found to be negative for mycoplasma contamination. A commercial iPS cell (WTC11) was used as a positive control (Coriell Institute, Camden, NJ).

**Differentiation of Human iPSCs into Induced Hepatocytes (iHep)**

Our hepatocyte differentiation protocol was reported by Collin de l'Hortet *et al*., 2019 (21). Briefly, human iPSCs were passaged with Accutase (Stem Cell Technologies, Vancouver, Canada) and re-plated at a density of 1 to 2x10^5^ per cm^2^ in reduced growth factor Matrigel (Corning Incorporated, Corning, NY)-coated plates in mTeSR. The next day, cells were exposed to a defined differentiation medium containing RPMI (Invitrogen, Carlsbad, CA), 1x B-27 without insulin supplement (Invitrogen, Carlsbad, CA), 0.5% penicillin/streptomycin (Millipore, Billerica, MA), 0.5% Non-Essential Amino Acids (Millipore, Billerica, MA), 100 ng/mL Activin A (R&D Systems, Minneapolis, MN), 10 ng/mL BMP4 (R&D Systems, Minneapolis, MN), and 20 ng/mL FGF2 (BD, Franklin Lakes, NJ) for two days and placed in a normal O_2_ incubator (endoderm induction). Cells were subsequently maintained in a similar medium without FGF2 and BMP4 for two days in ambient O_2_ (definitive endoderm). Finally, cells were grown for 10 days in a defined medium containing 45% DMEM low glucose (ThermoFisher Scientific, Waltham, MA), 45% F-12 (ThermoFisher Scientific, Waltham, MA), 10% CTS Knockout SR Xenofree Medium (ThermoFisher Scientific, Waltham, MA), 0.5% Non-Essential Amino Acids (ThermoFisher Scientific, Waltham, MA), 0.5% L-glutamine (ThermoFisher Scientific, Waltham, MA), 50 ng/mL HGF (R&D Systems, Minneapolis, MN), and 1% DMSO (Sigma-Aldrich, St. Louis, MO). The medium was changed every other day (hepatic specification).

**Genotyping and Sanger Sequencing**

Genotyping and Sanger sequencing were performed by extracting genomic DNA with the DNeasy Blood & Tissue Kit (QIAGEN, Hilden, Germany) following the manufacturer’s instructions. DNA samples were genotyped using TaqMan SNP genotyping assays for TM6SF2 rs58542926, PNPLA3 rs738409, GCKR rs780094, MBOAT7 rs62641738, HSD17B13 rs72613567, and MTARC1 rs2642438 (ThermoFisher Scientific, Waltham, MA). Amplification and genotype clustering were performed using a StepOnePlus system (Applied Biosystems, Foster City, CA).

For sequencing, polymerase chain reaction (PCR) amplification was conducted with the KOD ONE PCR Master Mix (Toyobo, Osaka, Japan) using the forward (CAAGATGTCCAGCCAGAGAGG) and reverse primers (CTTTCTTGTGACAAAGGAGAACCT) for *TM6SF2*. After DNA samples were amplified, the result of the amplification was confirmed with a 2% agarose gel. PCR products were then purified using the ExoSAP-IT Express PCR Cleanup Kit (Applied Biosystems, Foster City, CA) and sequenced at the Genomics Research Core at the University of Pittsburgh. Sequencing buffer and a 1:4 dilution of BigDye 3.1 (ThermoFisher Scientific, Waltham, MA) were added, and thermocycling was performed according to ABI recommendations. Unincorporated sequencing reagents were removed using CleanSeq magnetic beads (Agencourt, Beckman Coulter, Brea, CA) according to manufacturer’s instructions. Two control samples were included with every sequencing run to ensure the proper performance of reagents and equipment.

**Gene Editing**

The single-guide RNA (sgRNA) sequence (GCAAATACAGCTCCGAGATC) was designed to cut the human TM6SF2 gene at position chr19:379,549 and replace the major allele (C) with the minor allele (T). The sgRNA was cloned into a plasmid vector and nucleofected into the iPSC-TM6SF2-WT together with the donor DNA (ACAGATGTCCAGCAGGGTTCTGGCATGGCTGATGCCCTCTCTCCTGCACCATGGAAGGCAAATACAGCTCCAAGATCAGACCTGCCTTCTTCCTCACCATCCCCTACCTGCTGGTGCCATGCTGGGCTGGCATGAAGGTCT), using the Amaxa 4D-Nucleofector (Lonza, Walkersville, MD). For each nucleofection, 1 million cells were resuspended in 20 μL of the Amaxa NHDF Nucleofector Kit (Lonza, Walkersville, MD), containing 1 μg of each of the gRNA and donor plasmid vectors (ABM, Richmond, Canada). The plasmid vector containing the gRNA also included a puromycin resistance gene. Forty-eight hours after transfection, selection was performed using 1 μg/mL of puromycin. After 7 days of selection, single clones were harvested. DNA from selected clonal colonies was extracted and amplified before performing Sanger sequencing. Minor homozygous clones were identified, expanded, and cryopreserved, and one clone was used to perform the experiments.

Off-target experiments were performed using the same gRNA. For these, we used HepG2 cells which were seeded at a density of 0.3 × 10^5^ cells per well in 1000 μL of growth medium in 12-well plates one day prior to transfection. This density was targeted to allow the cells to reach 50–70% confluence at the time of transfection. On the day of transfection, 50 μL of Opti-MEM medium was added to a sterile 1.5 mL Eppendorf tube, followed by 3 μL of Lipofectamine 3000 reagent (ThermoFisher Scientific, Waltham, MA). This mixture was briefly vortexed. Subsequently, the Lipofectamine solution was combined with a solution of 4 μg of the plasmid containing Cas9 and TM6 gRNA and 2 μL of P3000 reagent (ThermoFisher Scientific, Waltham, MA) in 50 μL of Opti-MEM medium. This final mixture was incubated at room temperature for 15 minutes to allow DNA-lipid complexes to form before being added to the cells. Selection with 2 μg/mL puromycin commenced 48 hours post-transfection. Cells were harvested 72 hours after selection to conduct genome modification assays using the GeneArt Genomic Cleavage Detection Kit (ThermoFisher Scientific, Waltham, MA), according to the manufacturer's instructions. The gRNA sequence targeting the TM6SF2 locus was 5'-GCAAATACAGCTCCGAGATC-3'. After transfection and selection, cells were lysed in 50 μL of cell lysis buffer. The lysate was treated with proteinase K for 15 minutes at 68°C, followed by a denaturation step at 95°C for 10 minutes. Subsequently, 2 μL of the lysate were used for PCR amplification with the AmpliTaq Gold 360 Master Mix (ThermoFisher Scientific, Waltham, MA). Primers were designed for three regions of the gene locus: pre-mutation site, mutation target, and post-mutation site. The primers used were:

Pre-mutation: Forward 5'-ACAGCTATGTGGTGGGCTTC-3', Reverse 5'-CCCTGTTGTCCCTTCCATCC-3'

Mutation target: Forward 5'-GCAATCCACCTGCCTCATCA-3', Reverse 5'-CCCCGTGTCAGTTGCTTTTG-3'

Post-mutation: Forward 5'-GCCTATGCTCTCACCTTCCC-3', Reverse 5'-GCTGGATGCTGAAGGCTTTG-3'

The PCR conditions were as follows: an initial denaturation at 95°C for 3 minutes, followed by 40 cycles of 30 seconds at 95°C, 30 seconds at 55°C, and 30 seconds at 72°C. The final extension was at 72°C for 5 minutes. One and a half μL of PCR product were mixed with 1 μL of 10x Detection Reaction Buffer and 5 μL of water, then denatured and re-annealed using a thermal cycle: 95°C for 5 minutes, 4°C for 5 minutes, 37°C for 5 minutes, and finally, 4°C for 5 minutes. One μL of 10x detection enzyme was added, and the samples were incubated for 1 hour at 37°C. Digestion products were analyzed by electrophoresis on a 2% agarose gel.

**Embryoid Body Formation**

Embryoid bodies (EBs) were formed by plating iPSC-TM6SF2-WT and iPSC-TM6SF2-E167K cells at a density of 2.5x10^4^ cells per cm^2^ on low-attachment 6-well plates in mTeSR with 20% FBS and cultured at 37°C and 5% CO_2_. The medium was changed every 72 hours. EBs started to form in suspension after one week of culture. At day 20, EBs were fixed in 4% paraformaldehyde (PFA) for 24 hours and 70% ethanol overnight at 4°C, then embedded in paraffin. Five-micron sections were placed on glass slides and used for immunostaining of the three germ layers.

**Quantitative Real-Time PCR**

Total RNA was isolated from human cells using RNeasy Mini Kits (QIAGEN, Hilden, Germany) and reverse transcribed using Super-Script III (Invitrogen, Carlsbad, CA) following the manufacturer’s instructions. We performed qPCR with a StepOnePlus system (Applied Biosystems, Foster City, CA) using TaqMan Fast Advanced Master Mix (Life Technologies, Waltham, MA). The primers used are listed in Supplementary Table 2. Relative gene expression was normalized to ß-actin (ACTB) mRNA and mtDNA, using ∆∆CT method. Genomic DNA was extracted with the DNeasy Blood & Tissue Kit (QIAGEN, Hilden, Germany) following the manufacturers’ instructions. mtDNA content was analyzed by Sybr green qPCR using primers amplifying mitochondrial cytochrome b (CYB), mitochondrial cytochrome c oxidase subunit 1 (CO1), mitochondrial cytochrome c oxidase subunit 3 (CO3), and ATP synthase subunit a (ATP6).

**Immunostaining**

The samples were fixed with 4% PFA, washed for 15 minutes and washed 3 times with PBS. Following fixation, samples were washed 3 times with wash buffer (PBS, 0.1% BSA, and 0.1% TWEEN 20) for 5 minutes and then blocked and permeabilized in blocking buffer (PBS, 10% normal donkey or goat serum, 1% BSA, 0.1% TWEEN 20, and 0.1% Triton X-100) for 1 hour at room temperature. Subsequently, the samples were then incubated with primary antibodies in blocking buffer overnight at 4°C. The following day, samples were washed 3 times with wash buffer for 5 minutes and incubated with secondary antibodies in blocking buffer for 2 hours in the dark at room temperature. Samples were washed 3 times with wash buffer for 5 minutes, followed by 3 washes with PBS, and counterstained with 1 µg/mL of DAPI (Sigma Aldrich, St. Louis, MO) for 1 minute at room temperature in the dark. Finally, samples were washed 3 times with PBS and stored in the dark at 4°C. Samples were imaged using an Eclipse Ti inverted microscope (Nikon, Melville, NY) and the NIS-Elements software platform (Nikon, Melville, NY). Images were analyzed using ImageJ software. RGB stacks were generated, preprocessed to equalize the illumination within the stack, thresholded, and measured.

To better understand the role of TM6SF2 rs58542926 in ESLD tissue and cells, we first validated the TM6SF2 primary antibody. A substantial body of literature shows variability in antibody performance and the methodologies employed have inconsistent and frequently conflicting results (2, 3, 4). To understand the distribution of TM6SF2 in liver tissue, we analyzed ESLD tissue from patients that were WT (CC) or possessed the E167K (TT) for TM6SF2 rs58542926 (Supplementary Figure 1B). See Supporting Materials and Methods for details.

For endoplasmic reticulum (ER) and Golgi staining, the Revvity Phenix Opera High Content Imaging system was used at 40X/0.75 hNA in 6-well plates. The analysis was performed using Revvity Harmony 5.1 software to segment each marker using Revvity’s proprietary building blocks for finding cell nuclei and imaging regions such as the Golgi and ER. The data consisting of single cell level morphological measures (Golgi Area (µm^2^), ER Area (µm^2^), ER Intensity (AU)) were exported and Python libraries, pandas, and seaborn were used to perform transformations and visualizations. All antibodies used are listed in Supplementary Table 3.

For TM6SF2 immunohistochemistry staining, 5-7-micron sections were deparaffinized with xylene and dehydrated with ethanol. Antigen unmasking was performed by boiling in 10 mM citrate buffer, pH 6.0. After antigen unmasking, the slides were exposed to 3% hydrogen peroxide and incubated overnight at 4°C with the primary antibody. On the following day, tissue sections were incubated with the secondary biotinylated antibody corresponding to the animal species of the primary antibody (BA-1000; Vector Laboratories, Burlingame, CA) and exposed to 3,30-diaminobenzidine (SK-4105; Vector Laboratories) to visualize the peroxidase activity. Counterstaining was performed with Richard-Allan Scientific Signature Series Hematoxylin (Thermo Scientific, Waltham, MA). Samples were imaged using an Axiovert 40 CFL (Zeiss, NY, USA) microscope and the Zeiss Zen 3.8 software platform (Zeiss, NY, USA). All antibodies used are listed in Supplemental Table 3.

**Enzyme-Linked Immunosorbent Assay (ELISA)**

ELISA for ApoB100 was done using the ApoB100 ELISA Kit (Thermo Scientific, Waltham, MA) according to the manufacturer’s protocol. The quantity of extracellular VLDL was measured using the Biomatik Corporation Human Very Low-Density Lipoprotein (VLDL) Elisa Kit (Biomatik Corporation, Kitchener, Canada) according to the manufacturer’s instructions. The reaction was developed for 30 minutes with 100 μL/well TMB substrate solution and stopped with 50 μL/well stop solution. HRP activity was measured in an HTX microplate reader (Biotek, Winooski, VT) at a wavelength of 450 nm. To calculate the sample value, the absorbance was interpolated with a standard curve generated using a four-parameter algorithm.

**Western Blotting**

Human samples were incubated with RIPA lysis buffer (Sigma Aldrich, St. Louis, MO), 1x Halt™ Protease (Thermo Scientific, Waltham, MA), and Phosphatase Inhibitor Cocktail (Thermo Fisher Scientific, Waltham, MA) for 30 minutes at 4°C. Samples were centrifuged at 13,000 x g for 10 minutes at 4°C. The supernatant from each sample was then transferred to a new microfuge tube and used as the whole cell lysate. Protein concentrations were determined by comparison with a known concentration of bovine serum albumin using a Pierce BCA Protein Assay Kit (Thermo Fisher Scientific, Waltham, MA). Thirty µg of lysate were loaded per lane into 10% Mini-PROTEAN TGX™ gel (BioRad, Hercules, CA). Next, proteins were transferred onto the PVFD transfer membrane (Thermo Fisher Scientific, Waltham, MA). Membranes were incubated with a primary antibody solution overnight and then washed. Membranes were incubated for 1 hour in a secondary antibody solution and then washed. Target antigens were finally detected using SuperSignal™ West Pico PLUS Chemiluminescent Substrate (Thermo Fisher Scientific, Waltham, MA). Images were scanned and analyzed using ImageJ software. All band density values were normalized to the band density for GAPDH. All antibodies used are listed in Supplementary Table 3.

**Human Cytokine Antibody Array**

The Human Cytokine Antibody Array (Abcam, Cambridge, UK) was used for the simultaneous detection of cytokines and chemokines in cellular supernatants, according to the manufacturer’s recommendations. Briefly, 1 mL of cell cultured supernatant was added to the membranes and incubated overnight at 4°C on a rocking platform shaker. The membranes were then washed and incubated in biotin-conjugated anti-cytokines. HRP-conjugated streptavidin was added to the arrays and incubated for 2 hours at room temperature. The membrane arrays were developed with chemiluminescence detection reagents, and images were scanned and analyzed using ImageJ software. All band density values were normalized to the band density for the positive control on each membrane.

**Nile Red Staining**

Samples were fixed with 4% PFA for 15 minutes and washed three times with PBS. After that, the samples were incubated with a 0.3 mM Nile Red (Sigma Aldrich, St. Louis, MO) solution for 30 minutes at room temperature. Then, they were washed twice with PBS and counterstained with 1 µg/mL of DAPI (Sigma Aldrich, St. Louis, MO) for 1 minute. Samples were imaged using an Eclipse Ti inverted microscope (Nikon, Melville, NY) and the NIS-Elements software platform (Nikon, Melville, NY). Following that, images were analyzed using ImageJ software.

**Cholesterol Analysis**

For analysis of cholesterol metabolism, cells were cultured for 48 hours, after which the supernatant and cells were collected. The Qquantification of intracellular total cholesterol and its fractions was measured using the Cholesterol Assay Kit (Abcam, Cambridge, UK) according to the manufacturer’s instructions. The fluorescence signal (Ex/Em: 535/587 nm) was measured on an HTX microplate reader (Biotek, Winooski, VT).

**Transmission Electron Microscopy**

Fixed samples were processed by the Center for Biologic Imaging at the University of Pittsburgh Image acquisition was performed using either the JEM-1011 or the JEM- 1400Plus transmission electron microscopes (Jeol, Peabody, MA) at 80 kV fitted with a side mount AMT 2k digital camera (Advanced Microscopy Techniques, Danvers, MA). See Supporting Materials and Methods for details.

**Caspase Assay**

Caspase-3 activity was measured using the EnzChek Caspase-3 Assay Kit II (Thermo Fisher Scientific, Waltham, MA). Briefly, 50 μl of the supernatant and 50 μl of the working substrate (5 μM Z-DEVD-R110) were added to an individual well of a 96-well microplate and incubated for 30 minutes, according to the manufacturer’s instructions. The fluorescence signal (Ex/Em: 496/520 nm) was measured in an HTX microplate reader (Biotek, Winooski, VT). Caspase-3 activity was expressed as arbitrary units of fluorescence normalized by the cell number.

**Insulin-Resistance Response**

For measurement of the insulin-resistance response, iHep-TM6SF2-WT and iHep-TM6SF2-E167K were washed 3 times in PBS and incubated at 37°C for 3 hours in glucose-free starvation media with or without 100 nM insulin. Cells were harvested, centrifuged for 5 minutes at 300 x g, the supernatant discarded, and the cell pellet was stored at -80°C for further analysis.

**Reactive Oxygen Species Assay**

Cells were plated in a 12-well plate. Total reactive oxygen species (ROS) in live cells was measured using the Cellular ROS Assay Kit (Abcam, Cambridge, UK). Following the manufacturer’s instructions, the fluorescence (Ex/Em = 520/605) was quantitatively measured on a synergy HTX microplate reader (Biotek, Winooski, VT). ROS was expressed as arbitrary units of fluorescence normalized by the cell number.

**Total NAD/NADH Quantification**

Cells were plated on a 12-well plate, treated with 100 μM of palmitic acid, and collected 48 hours later. Nontreated cells were used as a control. Total NAD/NADH in live cells was measured using the NAD/NADH Assay Kit (Abcam, Cambridge, UK) following the manufacturer’s instructions. Fluorescence (Ex/Em = 540/590) was measured on a synergy HTX microplate reader (Biotek, Winooski, VT). Total NAD/NADH was expressed as arbitrary units of fluorescence normalized by the cell number.

**Fatty Acid Uptake Assay**

Fatty acid uptake was measured using the Fatty Acid Uptake Assay Kit (Abcam, Cambridge, UK) according to the manufacturer’s instructions. Briefly, iHep-TM6SF2-WT and iHep-TM6SF2-E167K were washed 3 times in PBS and incubated at 37°C for 1 hour in glucose-free starvation media. Following serum starvation, cells were treated with 10 μL of 10X test compound working solution in each well, and the plates were incubated at 37°C for 30 minutes. The 2X solution of quenched Uptake Reaction Mix was prepared by adding 200 μL of the 100X Extracellular Quenching Solution stock and 100 μL of the 200X Fluorescent Fatty Acid Probe to 9.7 mL of prewarmed Fatty Acid Uptake Assay Buffer. Next, 100 μL of prewarmed 2X Uptake Reaction Mix were added to all wells, and fluorescence measurements began immediately of all wells in kinetic mode at 37°C every 15 minutes for a total of 60 minutes. The fluorescence signal (Ex/Em = 488/523 nm) was measured using an HTX microplate reader (Biotek, Winooski, VT). Fatty acid uptake was expressed as arbitrary units of fluorescence normalized by the cell number.

**Fatty Acid Synthesis Assay**

For general metabolite extraction, cells were treated with 13C L-Glucose tracer (Sigma Aldrich, St. Louis, MO) at a final concentration of 7 mM for 24 hours. Lipid metabolites were recovered and the samples were dried under nitrogen for 15 minutes and either stored at -80°C or derivatized immediately. After treatment of cells with isotope tracers, cells were quenched with 300 µL of ice-cold optima-grade methanol (ThermoFisher Scientific, Waltham, MA). Subsequently, 300 µL of optima-grade water (ThermoFisher Scientific, Waltham, MA) containing 1 µg of norvaline (ThermoFisher Scientific, Waltham, MA) were added to each well. The cells were scraped over ice, and the contents transferred to 1.5 mL tubes (Eppendorf, Hamburg, Germany). Six hundred µL of optima-grade chloroform (ThermoFisher Scientific, Waltham, MA) were added to each tube. The tubes were vortexed at 4°C for 30 minutes before being centrifuged at 17,000 rcf for 15 minutes. After centrifugation, the mixture clarified into a polar supernatant and a non-polar supernatant containing polar and lipid metabolites, respectively.

Lipid metabolites were recovered in the lower chloroform layer utilizing the methodology described above. After recovery, the samples were dried under nitrogen for 15 minutes and either stored at -80°C or derivatized immediately. For derivatization, 500 µL of a solution consisting of 2% v/v H_2_SO_4_ (Sigma Aldrich, St. Louis, MO) in optima-grade methanol were added to each sample. The samples were incubated while shaking at 175 rpm at 50°C for 2 hours. After incubation, the reaction was dried through the addition of 100 µL of a saturated NaCl solution (ThermoFisher Scientific, Waltham, MA) in optima-grade water. To extract the fatty acid methyl esters (FAMEs), 500 µL of HPLC-grade hexane (ThermoFisher Scientific, Waltham, MA) was added to each sample and briefly vortexed, resulting in a phase separation into two layers. The hexane supernatant containing the FAMEs was transferred to a new tube, and dried under nitrogen for 15 minutes. Afterwards, the samples were reconstituted in 100 µL of MS-grade hexane and transferred to glass inserts for analysis using GC-MS. The samples were analyzed using a Select FAME column (Agilent, Santa Clara, CA). The temperature gradient for analysis of fatty acids consisted of an initial temperature of 80°C, a gradient of 20°C/min up to 170°C, a gradient of 1°C/min to 204°C, a gradient of 20°C/min up to 250°C, followed by a final hold at 250°C for 10 minutes. The total run time of the method was approximately 51 minutes.

**Lipidomics**

For analysis of lipidomics, iHep-TM6SF2-WT and iHep-TM6SF2-E167K cells were collected. Samples were thawed on ice, and 100 μL of ultrapure water extract was added to resuspend the cells. Approximately 10 mg of each sample was weighed and homogenized by ball-mill in 1 mL of extraction solution (MTBE:Methanol = 3:1, V/V, Merck, Darmstadt, Germany) with internal standards. The mixture was vortexed for 15 minutes. Next, 200 μL of water was added to the mixture (Millipore, Billerica, MA), vortexed for 1 minute, and incubated at 4°C for 10 minutes. After centrifugation at 12000 rpm for 10 minutes (4°C), 200 μL of the upper phase was collected for complete solvent drying at 20°C. The residue was reconstituted using 200 μL of reconstitution solution (ACN:IPA = 1:1, V/V, Merck, Darmstadt, Germany), followed by vortex for 3 minutes and centrifugation at 12000 rpm for 3 minutes. One hundred and twenty μL of the final supernatant was used for LC-MS analysis.

The sample extracts were analyzed using an LC-ESI-MS/MS system (UPLC, Nexera LC-40; MS, Triple Quad 6500+). The analytical conditions were as follows, UPLC: column, Thermo Accucore™C30 (2.6 μm, 2.1 mm×100 mm i.d.); solvent system, A: acetonitrile/water (60/40,V/V, 0.1% formic acid, 10 mmol/L ammonium formate), B: acetonitrile/isopropanol (10/90 VV/V, 0.1% formic acid, 10 mmol/L ammonium formate); gradient program, A/B (80:20, V/V) at 0 minutes, 70:30 V/V at 2.0 minutes, 40:60 V/V at 4 minutes, 15:85 V/V at 9 minutes, 10:90 V/V at 14 minutes, 5:95 V/V at 15.5 minutes, 5:95 V/V at 17.3 minutes, 80:20 V/V at 17.3 minutes, 80:20 V/V at 20 minutes; flow rate, 0.35 mL/min; temperature, 45°C; injection volume: 2 μL. The effluent was alternatively connected to an ESI-triple quadrupole-linear ion trap (QTRAP)-MS. Differential analysis was performed using MetwareBio’s bioinformatics pipeline.

**RNA-seq, Differential Gene Analysis, and Gene Set Enrichment Analysis (GSEA)**

Whole-genome strand-specific RNA-seq was used to profile RNA expression levels in iHep-TM6SF2-WT and iHep-TM6SF2-E167K. RNA-seq libraries were prepared as previously described (21). RNA was extracted using TRIzol, followed by column purification using Zymo RNA Clean and Concentrator Columns (Zymo, Irvine, CA) according to the manufacturer’s instructions. Total RNA was depleted of ribosomal RNA using pooled antisense oligo hybridization and depletion through RNaseH digestion. Following purification, first-strand complementary DNA (cDNA) was synthesized. Subsequently, second-strand cDNA was synthesized, purified, and fragmented. RNA-seq libraries were prepared using Illumina technology (Illumina DRAGEN RNA, 3.10.12). Briefly, end repair, A-tailing, and barcoded adapter ligation were followed by PCR amplification and size selection. The integrity of the libraries was confirmed by quBit quantification, fragment analyzer size distribution assessment, and Sanger sequencing of about 10 fragments from each library. Libraries were sequenced using paired-end Illumina sequencing.

RNA-seq data was processed using the R software (v.4.2.3) DESeq2 package. The adjusted *p*-value cutoff of 0.05 and logFC > 1.5 were used as filter criteria. The GSEA analysis was done using the R package WebGestaltR and GSEA software (Gene Set Enrichment Analysis, v4.3.2) from the Broad Institute. Gene collections were obtained from the MSigDB KEGG subset of canonical pathways.

Comparisons to population data were performed using the Kyoto Encyclopedia of Genes and Genomes (KEGG) Pathway Enrichment Analysis and overrepresentation analysis algorithm (ORA). The significantly altered pathways in our dataset were compared to those identified from the supplementary data of Prill et al., 2019 (22)*.* The initial dataset consisted of gene expression profiles from 116 wildtype patients and 9 E167K mutant carriers’ liver biopsies (22)*.* These results were further visualized with the R software (v.4.2.3) package ggplot2 (v.3.4.2).

**Transcription Profiling by the RT2 Profiler PCR Array**

Total RNA was isolated using the RNeasy Mini Kit (QIAGEN, Hilden, Germany) and reverse transcribed using SuperScript III (Invitrogen, Carlsbad, CA) to synthesize and amplify cDNA. Key genes involved in the regulation and enzymatic pathways of fatty liver were simultaneously assayed with the RT2 Profiler PCR Array Human Fatty Liver Assay (PAHS-157ZC-6, QIAGEN, Hilden, Germany) according to the manufacturer’s instructions and analyzed with the Data Analysis Center (QIAGEN Hilden, Germany). Ingenuity pathway analysis (IPA) was used to identify differentially expressed genes, predict downstream effects, and identify targets (QIAGEN Bioinformatics; www.qiagen.com/ingenuity). Regulatory effects analysis within IPA was used to identify the relationships between upstream regulators and biological functions.

**Transmission Electron Microscopy**

Human samples were briefly centrifuged and washed with a PBS solution. Samples were then fixed with 2.5% glutaraldehyde overnight at 4°C. Fixed samples were processed by the Center for Biologic Imaging at the University of Pittsburgh and treated with 1% osmium tetroxide and 1% potassium ferricyanide for 1 hour at room temperature. Samples were washed with PBS and dehydrated in a graded series of ethanol solutions (30%, 50%, 70%, and 90%—10 minutes each), followed by three 15-minute changes in fresh 100% ethanol. Infiltration was done with four 1-hour changes of EPON embedding plastic. The last change of EPON was allowed to polymerize overnight at 37°C and then for 48 hours at 60°C. Resin blocks were removed from the Eppendorf tubes, and 70 nm sections were placed onto copper TEM grids. Image acquisition was performed using either the JEM-1011 or the JEM- 1400Plus transmission electron microscopes (Jeol, Peabody, MA) at 80 kV fitted with a side mount AMT 2k digital camera (Advanced Microscopy Techniques, Danvers, MA).

**Statistical Analysis**

For statistical analysis, means between two groups were compared by *t* test. Since data for continuous variables were not normally distributed, p-values (p) were determined using an unpaired, two-tailed Welch’s t-test with 95% confidence. Data are reported as mean ± SD, and p-values ≤ 0.05 were considered statistically significant. Statistical analyses were performed using GraphPad Prism version 9.3.0.

**References:**

1) Collin de l'Hortet A, Takeishi K, Guzman-Lepe J, Morita K, Achreja A, Popovic B, Wang Y, et al. Generation of Human Fatty Livers Using Custom-Engineered Induced Pluripotent Stem Cells with Modifiable SIRT1 Metabolism. Cell Metab 2019;30:385-401 e389.

2) Yen YH, Kee KM, Li WF, Liu YW, Wang CC, Hu TH, Tsai MC, et al. Causes of Death among Patients with Hepatocellular Carcinoma According to Chronic Liver Disease Etiology. Cancers (Basel) 2023;15.

3) Mahdessian H, Taxiarchis A, Popov S, Silveira A, Franco-Cereceda A, Hamsten A, Eriksson P, et al. TM6SF2 is a regulator of liver fat metabolism influencing triglyceride secretion and hepatic lipid droplet content. Proc Natl Acad Sci U S A 2014;111:8913-8918.

4) Newberry EP, Hall Z, Xie Y, Molitor EA, Bayguinov PO, Strout GW, Fitzpatrick JAJ, et al. Liver-Specific Deletion of Mouse Tm6sf2 Promotes Steatosis, Fibrosis, and Hepatocellular Cancer. Hepatology 2021;74:1203-1219.
