# Supplementary material for: Human-induced pluripotent stem cell–based hepatic modeling of lipid metabolism–associated TM6SF2-E167K variant
Source: Hepatology. Author manuscript; Available in PMC 2025 Aug 23. (PMC11865362; doi:10.1097/HEP.0000000000001065)
Supplement: Supplementary file 1 [file NIHMS2022610-supplement-Sup_Fig_2.pdf]

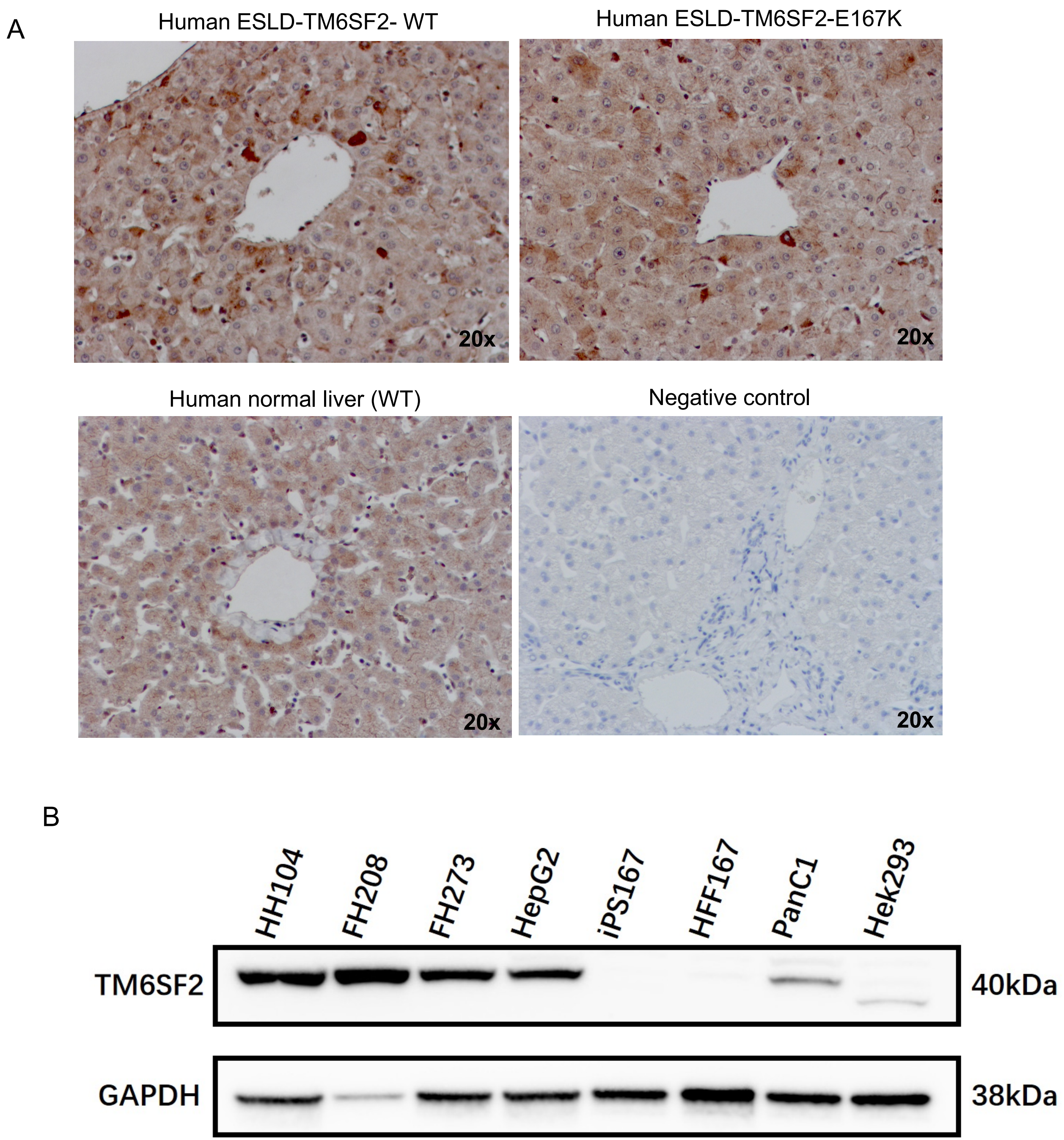
A
Human ESLD-TM6SF2- WT
Human ESLD-TM6SF2-E167K
20x
20x
Human normal liver (WT)
Negative control
20x
20x
B
HH104
FH208
FH273
HepG2
iPS167
HFF167
PanC1
Hek293
TM6SF2
40kDa
GAPDH
38kDa
