# Supplementary material for: Human-induced pluripotent stem cell–based hepatic modeling of lipid metabolism–associated TM6SF2-E167K variant
Source: Hepatology. Author manuscript; Available in PMC 2025 Aug 23. (PMC11865362; doi:10.1097/HEP.0000000000001065)
Supplement: Supplementary file 2 [file NIHMS2022610-supplement-Sup_Fig_3.pdf]

A

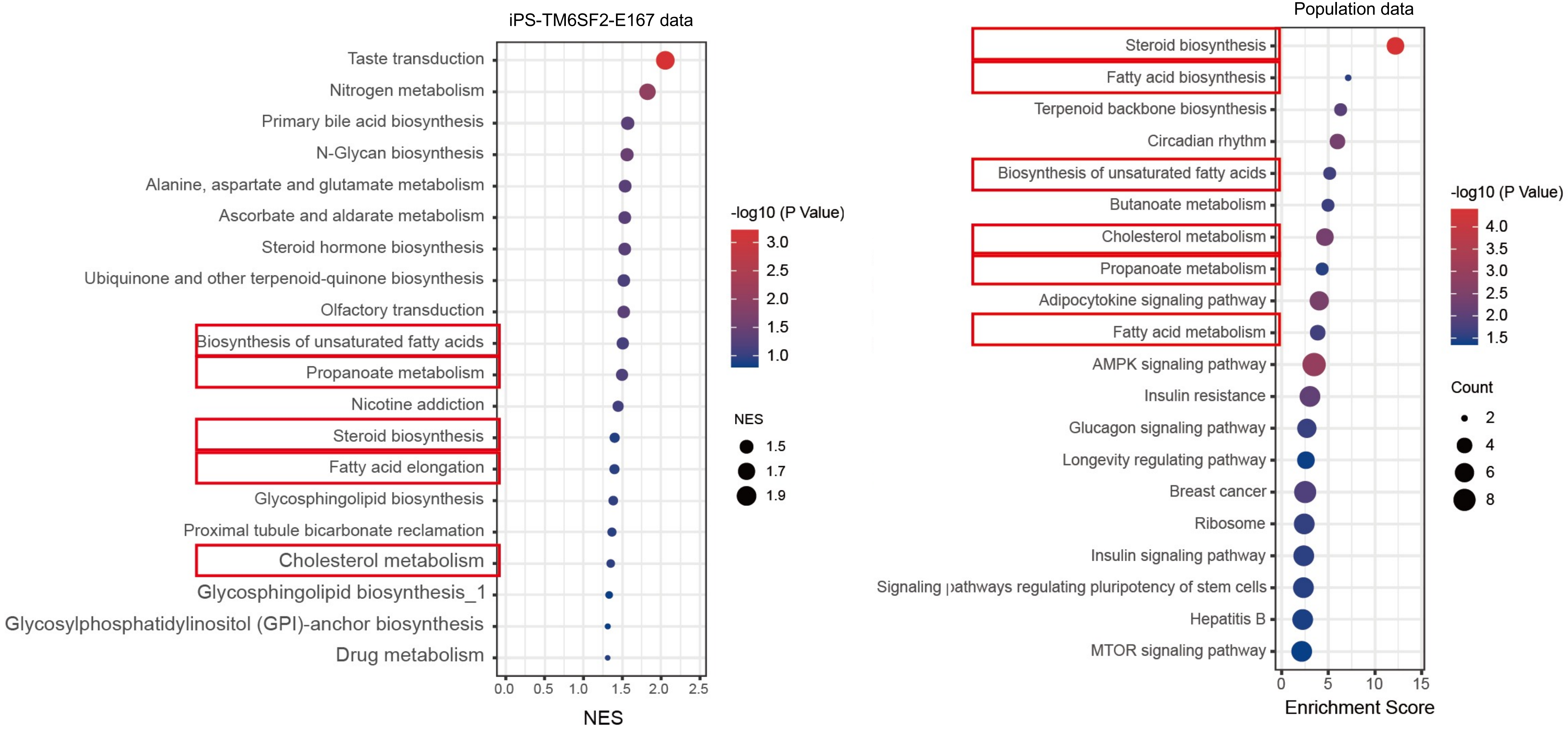
iPS-TM6SF2-E167 data
Taste transduction
Nitrogen metabolism
Primary bile acid biosynthesis
N-Glycan biosynthesis
Alanine, aspartate and glutamate metabolism
Ascorbate and aldarate metabolism
Steroid hormone biosynthesis
Ubiquinone and other terpenoid-quinone biosynthesis
Olfactory transduction
Biosynthesis of unsaturated fatty acids
Propanoate metabolism
Nicotine addiction
Steroid biosynthesis
Fatty acid elongation
Glycosphingolipid biosynthesis
Proximal tubule bicarbonate reclamation
Cholesterol metabolism
Glycosphingolipid biosynthesis_1
Glycosylphosphatidylinositol (GPI)-anchor biosynthesis
Drug metabolism
0.0 0.5 1.0 1.5 2.0 2.5
NES
-log10 (P Value)
3.0
2.5
2.0
1.5
1.0
NES
1.5
1.7
1.9
Population data
Steroid biosynthesis
Fatty acid biosynthesis
Terpenoid backbone biosynthesis
Circadian rhythm
Biosynthesis of unsaturated fatty acids
Butanoate metabolism
Cholesterol metabolism
Propanoate metabolism
Adipocytokine signaling pathway
Fatty acid metabolism
AMPK signaling pathway
Insulin resistance
Glucagon signaling pathway
Longevity regulating pathway
Breast cancer
Ribosome
Insulin signaling pathway
Signaling pathways regulating pluripotency of stem cells
Hepatitis B
MTOR signaling pathway
0 5 10 15
Enrichment Score
-log10 (P Value)
4.0
3.5
3.0
2.5
2.0
1.5
Count
2
4
6
8
