# Supplementary material for: Human-induced pluripotent stem cell–based hepatic modeling of lipid metabolism–associated TM6SF2-E167K variant
Source: Hepatology. Author manuscript; Available in PMC 2025 Aug 23. (PMC11865362; doi:10.1097/HEP.0000000000001065)
Supplement: Supplementary file 3 [file NIHMS2022610-supplement-Sup_Fig_4.pdf]

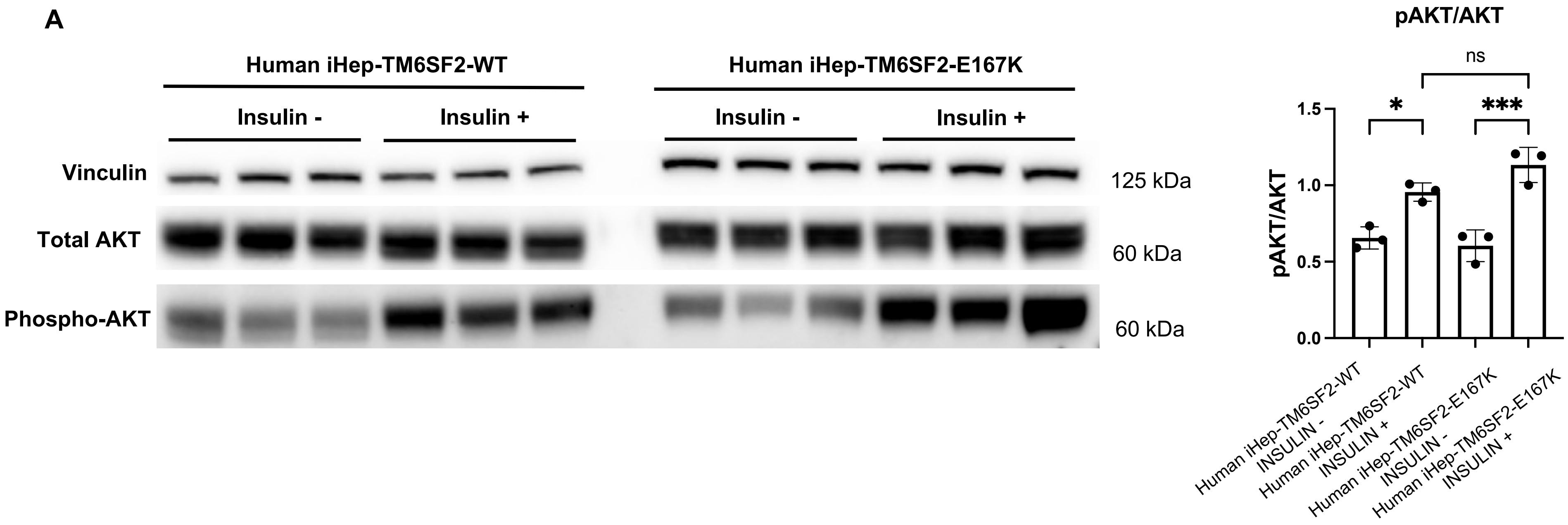
A
Human iHep-TM6SF2-WT
Human iHep-TM6SF2-E167K
Insulin -
Insulin +
Insulin -
Insulin +
Vinculin
Total AKT
Phospho-AKT
125 kDa
60 kDa
60 kDa
pAKT/AKT
ns
*
***
1.5
1.0
0.5
0.0
pAKT/AKT
Human iHep-TM6SF2-WT INSULIN -
Human iHep-TM6SF2-WT INSULIN +
Human iHep-TM6SF2-E167K INSULIN -
Human iHep-TM6SF2-E167K INSULIN +
