# Supplementary material for: Human-induced pluripotent stem cell–based hepatic modeling of lipid metabolism–associated TM6SF2-E167K variant
Source: Hepatology. Author manuscript; Available in PMC 2025 Aug 23. (PMC11865362; doi:10.1097/HEP.0000000000001065)
Supplement: Supplementary file 4 [file NIHMS2022610-supplement-Sup_Fig_5.pdf]

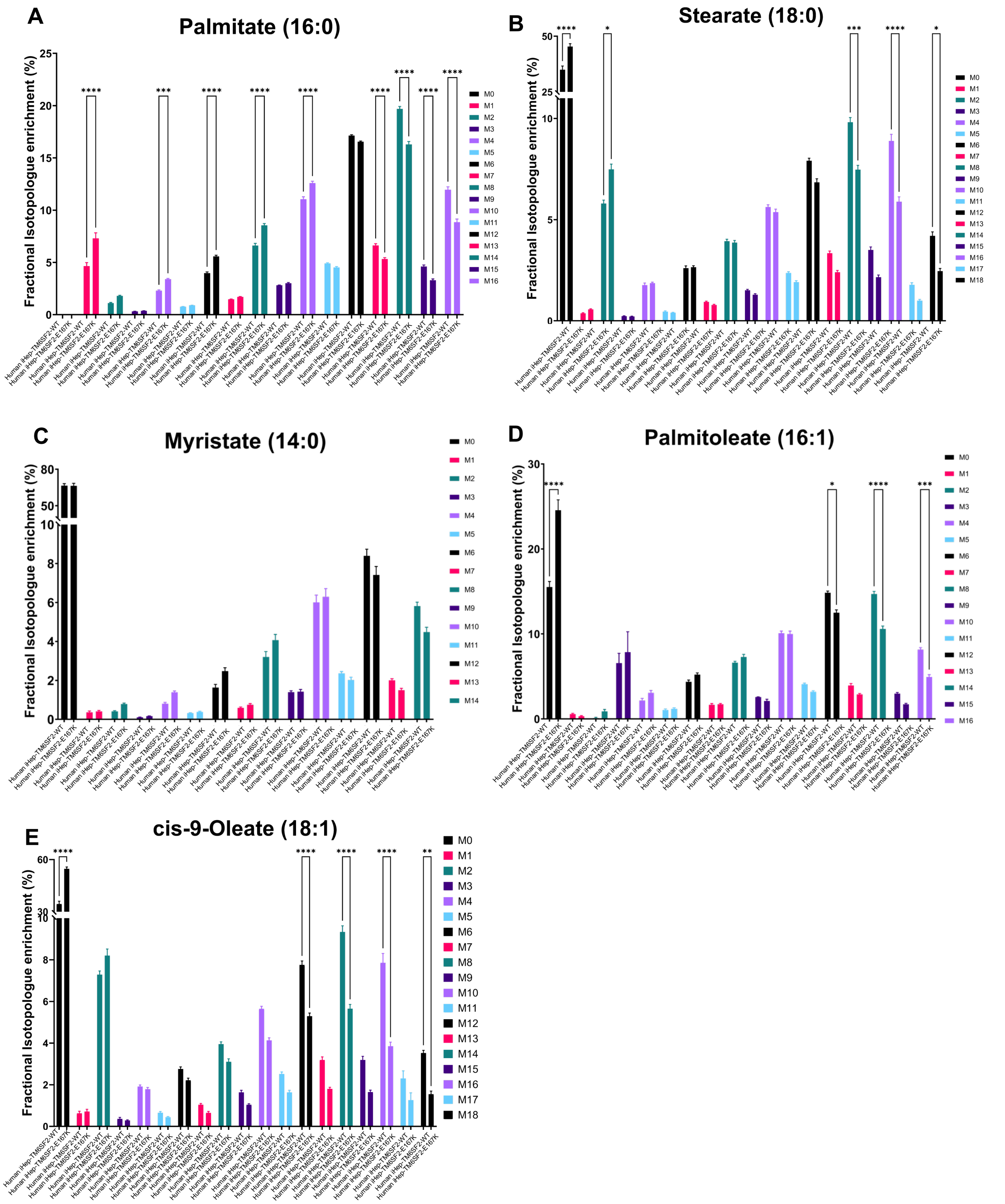
A
Palmitate (16:0)
Fractional Isotopologue enrichment (%)
0
5
10
15
20
25
****
***
****
****
****
****
****
****
****
M0
M1
M2
M3
M4
M5
M6
M7
M8
M9
M10
M11
M12
M13
M14
M15
M16
Human iHep-TM6SF2-WT
Human iHep-TM6SF2-E167K
B
Stearate (18:0)
Fractional Isotopologue enrichment (%)
0
5
10
25
50
****
*
***
****
*
M0
M1
M2
M3
M4
M5
M6
M7
M8
M9
M10
M11
M12
M13
M14
M15
M16
M17
M18
Human iHep-TM6SF2-WT
Human iHep-TM6SF2-E167K
C
Myristate (14:0)
Fractional Isotopologue enrichment (%)
0
2
4
6
8
10
50
80
M0
M1
M2
M3
M4
M5
M6
M7
M8
M9
M10
M11
M12
M13
M14
Human iHep-TM6SF2-WT
Human iHep-TM6SF2-E167K
D
Palmitoleate (16:1)
Fractional Isotopologue enrichment (%)
0
10
20
30
****
*
****
***
M0
M1
M2
M3
M4
M5
M6
M7
M8
M9
M10
M11
M12
M13
M14
M15
M16
Human iHep-TM6SF2-WT
Human iHep-TM6SF2-E167K
E
cis-9-Oleate (18:1)
Fractional Isotopologue enrichment (%)
0
2
4
6
8
10
30
60
****
****
****
****
**
M0
M1
M2
M3
M4
M5
M6
M7
M8
M9
M10
M11
M12
M13
M14
M15
M16
M17
M18
Human iHep-TM6SF2-WT
Human iHep-TM6SF2-E167K
