# Supplementary material for: Human-induced pluripotent stem cell–based hepatic modeling of lipid metabolism–associated TM6SF2-E167K variant
Source: Hepatology. Author manuscript; Available in PMC 2025 Aug 23. (PMC11865362; doi:10.1097/HEP.0000000000001065)
Supplement: Supplementary file 5 [file NIHMS2022610-supplement-Sup_Figure_1.pdf]

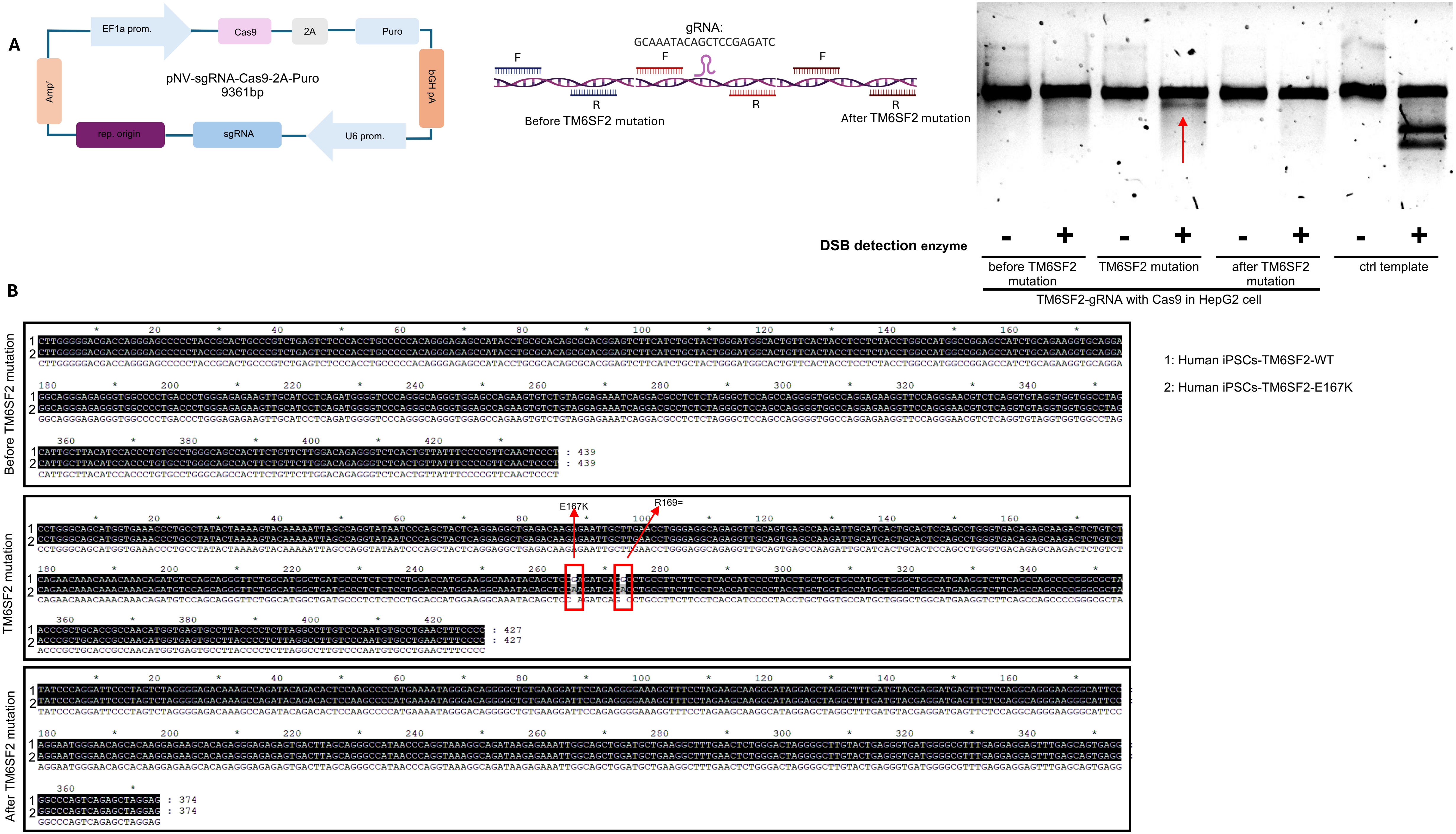
A
EF1a prom.
Cas9
2A
Puro
Ampr
pNV-sgRNA-Cas9-2A-Puro
9361bp
bGH pA
rep. origin
sgRNA
U6 prom.
gRNA:
GCAAATACAGCTCCGAGATC
F
R
Before TM6SF2 mutation
After TM6SF2 mutation
DSB detection enzyme
- + - + - + - +
before TM6SF2 mutation
TM6SF2 mutation
after TM6SF2 mutation
ctrl template
TM6SF2-gRNA with Cas9 in HepG2 cell
B
Before TM6SF2 mutation
TM6SF2 mutation
After TM6SF2 mutation
E167K
R169=
1: Human iPSCs-TM6SF2-WT
2: Human iPSCs-TM6SF2-E167K
