# Supplementary material for: Human-induced pluripotent stem cell–based hepatic modeling of lipid metabolism–associated TM6SF2-E167K variant
Source: Hepatology. Author manuscript; Available in PMC 2025 Aug 23. (PMC11865362; doi:10.1097/HEP.0000000000001065)
Supplement: Supplementary file 6 [file NIHMS2022610-supplement-Sup_Tab_1.pdf]

Supplementary table 1: Characteristics of human cells used in this study.

Tissue ID	Age	Gender	Viability (%)
Fetal Hepatocyte #208	22 weeks	Unknown	87%
Fetal Hepatocyte #273	19 weeks	Male	84%
Fetal Liver #167	16 weeks	Male	90%
Adult normal liver HH080	16 years	Male	80%
Adult normal liver HH104	11 years	Male	80%
Adult normal liver HH98	15 years	Male	85%
Adult liver ESLD #120	36 years	Female	80%
Adult liver ESLD #130	64 years	Female	85%
