# Supplementary material for: Human-induced pluripotent stem cell–based hepatic modeling of lipid metabolism–associated TM6SF2-E167K variant
Source: Hepatology. Author manuscript; Available in PMC 2025 Aug 23. (PMC11865362; doi:10.1097/HEP.0000000000001065)
Supplement: Supplementary file 7 [file NIHMS2022610-supplement-Sup_Tab_2.pdf]

Supplementary table 2. Taqman® Gene Expression Assay IDs used for qPCR and genotyping

Target gene	Gene Expression Assay ID	Company
Sox17	Hs00751752_s1	Life Technologies
Sox9	Hs01001343_g1	Life Technologies
HNF1ß	Hs01001602_m1	Life Technologies
ITPR3	Hs01573539_m1	Life Technologies
HNF4a	Hs00604431_m1	Life Technologies
UGT1A1	Hs02511055_s1	Life Technologies
LXR	Hs00172885_m1	Life Technologies
CFTR	Hs00357011_m1	Life Technologies
LRRC8A	Hs01555916_m1	Life Technologies
TMEM16A	Hs00216121_m1	Life Technologies
ß-actin	Hs01060665_g1	Life Technologies
BSEP	Hs00184824_m1	Life Technologies
MRP2	Hs00166123_m1	Life Technologies
ACC	Hs01046047_m1	Life Technologies
FASN	Hs01005622_m1	Life Technologies
SCTR	Hs01085380_m1	Life Technologies
AE2	Hs01586776_m1	Life Technologies
AQP1	Hs01028916_m1	Life Technologies
CHRM3	Hs00265216_s1	Life Technologies
P2Y1R	Hs00704965_s1	Life Technologies
c-Myc	Hs00153408_m1	Life Technologies

Sox2	Hs01053049_s1	Life Technologies
Nanog	Hs02387400_g1	Life Technologies
Oct3/4	Hs04260367_gH	Life Technologies
Lin28A	Hs00702808_s1	Life Technologies
HSP70	Hs00382884_m1	Life Technologies
FOXA1	Hs04187555_m1	Life Technologies
ELOVL6	Hs00907564_m1	Life Technologies
EGFR	Hs01076090_m1	Life Technologies
PPARA	Hs00947536_m1	Life Technologies
FOXA2	Hs00232764_m1	Life Technologies
Srebf1	Hs01088691_m1	Life Technologies
RXR	Hs00172885_m1	Life Technologies
PPARGC1A	Hs00173304_m1	Life Technologies
ACOX1	Hs01074241_m1	Life Technologies
CSS2	Hs00226041_m1	Life Technologies
VAMP7	Hs00194568_m1	Life Technologies
MERC	Hs00358796_g1	Life Technologies
APOH	Hs00909569_g1	Life Technologies
SULT1E1	Hs00960938_m1	Life Technologies
CD36	Hs00354519_m1	Life Technologies
XPB1	Hs00231936_m1	Life Technologies
TM6SF2 rs58542926	C__89463510_10	Life Technologies
PNPLA3 rs738409	C_______7241_10	Life Technologies
MBOAT7 rs62641738	C___8716820_10	Life Technologies

GCKR rs780094	C___2862873_10	Life Technologies
HSD17B13 rs72613567	ANWDFW4	Life Technologies
MTARC1 rs2642438	C___1235772_10	Life Technologies
