# Supplementary material for: Human-induced pluripotent stem cell–based hepatic modeling of lipid metabolism–associated TM6SF2-E167K variant
Source: Hepatology. Author manuscript; Available in PMC 2025 Aug 23. (PMC11865362; doi:10.1097/HEP.0000000000001065)
Supplement: Supplementary file 8 [file NIHMS2022610-supplement-Sup_Tab_3.pdf]

Supplementary table 3. Antibodies and dilutions used for Western blot and Immunofluorescence staining.

Target protein	Catalog number	Company	Dilution	Host
anti-Sox17	NL1924R	R&D	1:50	Goat
anti-HNF4α	Ab41898	Abcam	1:500	Mouse
anti-Alb	A80-229A	Bethyl	1:100	Goat
anti-AFP	ZSA06	Invitrogen	1:300	Mouse
anti-TRA-1-60	560218	BD Biosciences	1:10	Mouse
anti-TM6SF2	PA5-69304	Invitrogen	1:100	Rabbit
anti-HSPA5	NBP1-06274	Novus	1:1,000	Rabbit
anti-XBP1	Ab37152	Abcam	1:50	Rabbit
anti-OCT3/4	SC-9081	Santa-Cruz	1:250	Mouse
anti-SSEA4	14-8843-80	Invitrogen	1:10	Mouse
anti-Nanog	4893S	Cell signaling	1:2000	Rabbit
anti-SOX1	SC022	R&D	1:10	Goat
anti-Otx-2				Goat
anti-HAND-1				Goat
anti-Brachyury				Goat
anti-GATA-4				Goat
anti-SOX17				Goat
anti-TM6SF2 (WB)	PA5-69304	Invitrogen	1:1,500	Rabbit
Anti-ATF4 (WB)	10835-1-AP	Proteintech	1:1000	Rabbit
Anti-CHOP	15204-1-AP	Proteintech	1:100	Rabbit
Anti-GM130	GM130	BD	1:100	Mouse
Anti-Calnexin	HPA009433	sigma	1:100	Rabbit

Anti-Perilipin2	610102	Progen	1:100	Mouse
anti-HSP70 (WB)	Ab2787	Abcam	1:5,000	Mouse
anti-ApoB (WB)	SC-393636	Santa-Cruz	1:1,000	Mouse
anti-GAPDH (WB)	60004-1-1g	ProteinTech	1:10,000	Mouse
