# Supplementary material for: Human-induced pluripotent stem cell–based hepatic modeling of lipid metabolism–associated TM6SF2-E167K variant
Source: Hepatology. Author manuscript; Available in PMC 2025 Aug 23. (PMC11865362; doi:10.1097/HEP.0000000000001065)
Supplement: Supplementary file 9 [file NIHMS2022610-supplement-Sup_Tab_4.pdf]

Supplementary table 4: Up and down regulated genes analysed by RNA-seq

Up-regulted genes						
**Gene**	**BaseMean**	**log2FoldChange**	**lfcSE**	**stat**	**pvalue**	**padj**
ENSG00000010379.16	134.1086494	2.356323616	0.330962733	7.119604064	1.08E-12	2.01E-09
ENSG00000012504.15	20.93632437	2.933192572	0.688058074	4.263001459	2.02E-05	0.002758282
ENSG00000021826.16	13367.2312	1.782918849	0.499680875	3.568115048	0.000359559	0.020870055
ENSG00000037965.6	27.40056724	2.597604634	0.763384276	3.402748416	0.000667117	0.031260939
ENSG00000066279.18	376.8948899	1.712697988	0.449671906	3.8087725	0.000139658	0.010742628
ENSG00000074276.10	158.6956445	1.58316766	0.460104824	3.440884719	0.000579816	0.028437039
ENSG00000077943.8	218.3596198	2.550537556	0.729257781	3.497443047	0.000469741	0.025000524
ENSG00000082556.12	90.27568305	2.170798805	0.444657851	4.881953175	1.05E-06	0.000274505
ENSG00000084453.16	157.7459165	3.49077367	0.74096098	4.711143726	2.46E-06	0.000545783
ENSG00000091583.11	2152.189693	3.288573352	0.665212908	4.943640318	7.67E-07	0.000216229
ENSG00000100181.22	141.7242693	3.376941415	0.586493999	5.757844787	8.52E-09	5.60E-06
ENSG00000107807.13	23.78050428	2.715067846	0.669685806	4.054241288	5.03E-05	0.005001267
ENSG00000109339.23	201.3211682	1.622318418	0.477907932	3.394625426	0.000687226	0.031811437
ENSG00000110900.15	21.59918538	2.827511411	0.665219903	4.250491304	2.13E-05	0.002805129
ENSG00000111181.12	21.67017503	1.952902339	0.570083884	3.425640318	0.000613352	0.029417806
ENSG00000111339.12	15.31253871	2.467372233	0.742903952	3.321253343	0.000896142	0.03756932
ENSG00000112984.12	136.6413387	2.464386512	0.612963624	4.020444957	5.81E-05	0.005619843
ENSG00000113100.10	20.80886858	2.757357995	0.812223078	3.394828427	0.000686716	0.031811437
ENSG00000117724.13	374.4885183	1.616630128	0.436978186	3.69956712	0.000215968	0.014386542
ENSG00000118514.14	148.3319298	2.495820303	0.668307433	3.734539195	0.000188059	0.013128078
ENSG00000122966.16	227.5215979	1.751250839	0.354380885	4.941719242	7.74E-07	0.000216229
ENSG00000124564.17	33.0881882	2.472639695	0.523395968	4.724223807	2.31E-06	0.000519057
ENSG00000130226.17	6.520788416	5.809317046	1.392310872	4.17242813	3.01E-05	0.003590153
ENSG00000130649.10	24.97741883	2.117472237	0.528783558	4.004421479	6.22E-05	0.005949586
ENSG00000131620.17	318.5138556	1.902217934	0.500367218	3.801643806	0.000143739	0.010838664
ENSG00000131849.12	55.34913958	2.341205225	0.386226298	6.061744731	1.35E-09	1.06E-06
ENSG00000132872.12	26.27637121	3.705773321	0.904209137	4.098358632	4.16E-05	0.00448761
ENSG00000132874.14	28.53784202	1.966319066	0.553900351	3.549950929	0.000385303	0.021636407
ENSG00000132967.9	17.05369186	2.291505134	0.697161347	3.286907894	0.00101294	0.040565345
ENSG00000133800.9	40.42306975	3.277495031	0.99029527	3.309613939	0.000934247	0.038472116
ENSG00000135119.14	138.6795926	2.353382154	0.331744764	7.093954185	1.30E-12	2.18E-09

ENSG00000136011.15	25.18619765	4.6775075	1.240167581	3.771673743	0.000162156	0.011665041
ENSG00000137558.9	15.36560727	3.448033993	0.906732441	3.8027028	0.000143126	0.010838664
ENSG00000138792.10	395.6508988	1.508744165	0.449080604	3.359628877	0.000780472	0.034159866
ENSG00000139209.16	196.1187183	2.295301577	0.593945733	3.864497126	0.000111318	0.008864353
ENSG00000139304.16	14.25409904	2.843815731	0.837960558	3.393734592	0.000689465	0.031811437
ENSG00000139567.12	77.86002812	1.544155326	0.42063987	3.670967583	0.000241634	0.015736317
ENSG00000139973.16	157.935978	1.620101362	0.484815979	3.341683099	0.000832721	0.0354367
ENSG00000142677.4	199.9119008	1.889088394	0.370486117	5.098945167	3.42E-07	0.000118003
ENSG00000147246.10	10.61319949	3.887433249	1.149453967	3.381982543	0.000719647	0.032743947
ENSG00000148386.9	16.73036207	6.196386497	1.515913685	4.087558914	4.36E-05	0.004604207
ENSG00000149124.11	137.6270563	2.591795213	0.443115186	5.849032705	4.94E-09	3.70E-06
ENSG00000150636.17	43.71899581	1.504481598	0.469062219	3.207424381	0.001339293	0.048942961
ENSG00000151491.14	269.4629014	1.783317842	0.490505903	3.635670502	0.000277259	0.017389656
ENSG00000153253.18	20.52956541	2.988316965	0.80357884	3.718760146	0.000200203	0.013603129
ENSG00000155754.15	5.396592172	5.534037111	1.406337659	3.935069984	8.32E-05	0.007321666
ENSG00000156006.5	24.6688662	3.284588266	0.916515991	3.583776278	0.000338662	0.020236291
ENSG00000157152.17	201.538287	1.619658696	0.239419471	6.764941413	1.33E-11	1.94E-08
ENSG00000161031.13	130.795591	3.592505632	0.945434434	3.799846403	0.000144786	0.010846891
ENSG00000161381.14	40.17964868	1.801646657	0.538195365	3.347570001	0.000815234	0.034980242
ENSG00000161798.7	24.98529681	2.065978034	0.503959342	4.099493478	4.14E-05	0.00448761
ENSG00000162692.12	145.5770032	3.015395762	0.597337975	5.048056361	4.46E-07	0.000143951
ENSG00000165169.11	110.2434697	2.49494185	0.549690527	4.538811803	5.66E-06	0.001077725
ENSG00000165899.11	38.42455057	1.657389735	0.473620862	3.499401877	0.000466303	0.024947833
ENSG00000166278.15	264.3568962	1.54719727	0.392175365	3.945166903	7.97E-05	0.007079023
ENSG00000166415.15	300.4264757	2.03090912	0.303440127	6.692948415	2.19E-11	2.97E-08
ENSG00000166863.12	153.9825249	1.862273271	0.54701252	3.404443598	0.00066299	0.031256963
ENSG00000166866.13	187.7773921	1.775789215	0.401415011	4.423823636	9.70E-06	0.001594048
ENSG00000168509.20	22.19344236	3.172111733	0.706022054	4.492935758	7.02E-06	0.001256083
ENSG00000170373.8	17.95576664	3.289605224	1.016160435	3.237289222	0.00120671	0.045891018
ENSG00000171954.13	15.18044018	3.137465281	0.838370429	3.742337723	0.000182316	0.012820387
ENSG00000173535.14	95.49748286	1.539791839	0.377325333	4.080806945	4.49E-05	0.00464688
ENSG00000173578.7	13.58916449	3.507759956	1.007503991	3.481633808	0.000498365	0.025849024
ENSG00000173867.10	14.65692597	3.072962405	0.757807093	4.055072106	5.01E-05	0.005001267
ENSG00000175329.13	56.74254818	1.51184161	0.388423494	3.892250682	9.93E-05	0.008196393

ENSG00000178187.7	19.28102217	1.896492189	0.548711896	3.45626221	0.000547722	0.027567333
ENSG00000179542.16	156.0744471	2.84715374	0.734380342	3.876947105	0.000105775	0.008522232
ENSG00000180532.10	9.01200328	5.301961638	1.487578628	3.56415556	0.000365029	0.020926996
ENSG00000183114.8	26.70964588	1.805170025	0.562220342	3.210787462	0.001323718	0.048705176
ENSG00000184434.8	20.47798975	3.25858052	0.778399213	4.186258753	2.84E-05	0.003473872
ENSG00000185306.13	11.43801523	2.936840303	0.906597889	3.239407833	0.001197782	0.045722064
ENSG00000185666.14	60.66047157	2.15382895	0.663706674	3.245151865	0.00117388	0.04497814
ENSG00000185736.16	41.31009361	1.55655562	0.452543221	3.439573384	0.000582632	0.028437039
ENSG00000186094.17	16.57270089	3.62813083	0.739330128	4.90732177	9.23E-07	0.000247635
ENSG00000186204.15	60.19553614	5.590343564	0.699742965	7.989138644	1.36E-15	5.54E-12
ENSG00000186529.16	1967.120979	3.766608479	0.614279587	6.131749383	8.69E-10	7.09E-07
ENSG00000187510.9	27.38440335	3.31112426	0.616779174	5.368411257	7.94E-08	3.37E-05
ENSG00000189229.11	17.50520897	2.819984992	0.787020631	3.583114446	0.000339522	0.020236291
ENSG00000196090.12	668.2208998	5.601563909	0.41410316	13.52697696	1.08E-41	2.21E-37
ENSG00000198028.4	15.95349727	2.817919537	0.783587873	3.596175533	0.00032293	0.019649564
ENSG00000198300.14	37.86512244	8.346677367	1.321362332	6.316721131	2.67E-10	3.03E-07
ENSG00000203797.10	5.662484607	5.599128755	1.443496128	3.878866487	0.000104944	0.00848883
ENSG00000203799.13	183.441802	2.169481812	0.584878234	3.709287998	0.000207843	0.013936414
ENSG00000204613.11	6.252064175	4.739065098	1.393107979	3.401793091	0.000669453	0.031298464
ENSG00000204970.9	9.351439633	6.325305815	1.568054288	4.033856393	5.49E-05	0.005377148
ENSG00000206195.11	9.0706646	5.297425951	1.363563359	3.884987021	0.000102335	0.008344016
ENSG00000214429.3	6.702235293	5.852170123	1.585025125	3.692162369	0.000222355	0.014668262
ENSG00000214553.10	94.85092642	1.908179951	0.340463647	5.604651093	2.09E-08	1.22E-05
ENSG00000215808.4	45.70782802	3.482706009	0.810994794	4.294362964	1.75E-05	0.002498704
ENSG00000217330.1	53.2883736	7.039353328	1.274505589	5.523203185	3.33E-08	1.70E-05
ENSG00000224597.10	214.2024888	1.829395996	0.348231438	5.253391268	1.49E-07	6.09E-05
ENSG00000224957.6	29.69624505	3.877246056	0.954551018	4.061853145	4.87E-05	0.004961945
ENSG00000225431.1	34.23630058	2.240105746	0.535235391	4.185272092	2.85E-05	0.003473872
ENSG00000226453.2	18.57155866	2.64102932	0.711839212	3.710148691	0.000207138	0.013934957
ENSG00000226476.5	10.99314341	3.502865919	0.990456098	3.536619064	0.000405284	0.022267667
ENSG00000226674.11	35.5047516	3.28758106	0.504309678	6.518972771	7.08E-11	8.49E-08
ENSG00000227722.2	9.348581955	5.339408688	1.321127323	4.041554962	5.31E-05	0.005228735
ENSG00000229970.3	8.042661988	5.118089042	1.331475449	3.843922956	0.000121083	0.009529562
ENSG00000230453.9	30.34394002	1.85381807	0.498949081	3.715445407	0.000202846	0.013691437

ENSG00000233098.9	87.77521625	1.834777343	0.391538436	4.686072101	2.78E-06	0.000603926
ENSG00000233508.3	9.61175466	6.373106809	1.405817429	4.533381561	5.80E-06	0.00109558
ENSG00000234985.2	13.33022011	6.843316959	1.516696439	4.511988546	6.42E-06	0.001179383
ENSG00000236532.6	11.95214614	2.630349319	0.797767271	3.297138672	0.000976753	0.039504217
ENSG00000237978.6	9.592268949	3.350757894	1.007954682	3.324314033	0.000886363	0.037329811
ENSG00000239922.2	48.33830371	1.962989765	0.430625494	4.558461567	5.15E-06	0.000990926
ENSG00000240086.7	26.96762794	2.084427743	0.623415491	3.343561033	0.000827105	0.0353453
ENSG00000241644.2	1082.754535	2.031156249	0.531404665	3.822240153	0.000132245	0.010249726
ENSG00000242082.2	98.18858467	1.503954223	0.441817948	3.40401342	0.000664035	0.031256963
ENSG00000242781.2	4.495362146	5.269526169	1.448357123	3.638278217	0.000274467	0.017267692
ENSG00000244306.11	21.41709775	4.353374909	0.850365936	5.119413568	3.06E-07	0.000107714
ENSG00000244694.7	37.51727658	2.210140554	0.511649236	4.319640091	1.56E-05	0.002308471
ENSG00000245146.7	21.5668606	1.751100548	0.492200102	3.5577005	0.000374116	0.021124573
ENSG00000248740.6	15.79391246	4.612686294	1.00350717	4.596565358	4.30E-06	0.000893386
ENSG00000248869.7	9.352130134	6.327279922	1.328936864	4.761159158	1.92E-06	0.000445863
ENSG00000248874.5	11.30425006	6.601601957	1.326425091	4.976988147	6.46E-07	0.000193592
ENSG00000250159.7	61.02156887	3.586669581	0.570605185	6.28572904	3.26E-10	3.17E-07
ENSG00000250546.6	18.66440435	2.950140426	0.69516567	4.243794755	2.20E-05	0.002864537
ENSG00000250616.2	34.98637454	3.327855761	0.529309072	6.287169324	3.23E-10	3.17E-07
ENSG00000250682.6	25.20388336	1.904271388	0.557911429	3.413214516	0.000642014	0.030576659
ENSG00000251095.7	156.4955109	1.989545833	0.561317949	3.544418697	0.00039348	0.021854749
ENSG00000251138.7	87.53733281	2.039771885	0.472681872	4.315316507	1.59E-05	0.002337185
ENSG00000251209.9	32.80850788	2.635687644	0.617536495	4.268067825	1.97E-05	0.002734138
ENSG00000251226.1	23.42254209	1.642072475	0.511976708	3.207318712	0.001339785	0.048942961
ENSG00000253706.5	13.58208067	4.937925046	1.149894647	4.294241268	1.75E-05	0.002498704
ENSG00000254101.7	5.169376438	5.475516844	1.607607061	3.406004475	0.000659211	0.031177162
ENSG00000254245.2	29.0719355	4.078389471	0.742502392	5.492762734	3.96E-08	1.88E-05
ENSG00000254349.6	5.523439251	4.581163589	1.430804753	3.201809037	0.001365675	0.049374435
ENSG00000255282.6	7.061552534	3.934400066	1.223927289	3.214570098	0.0013064	0.048329692
ENSG00000256040.2	21.07387462	2.273091838	0.630018489	3.607976395	0.000308595	0.019061792
ENSG00000257185.2	3.984052103	5.101919851	1.522048937	3.352007762	0.000802278	0.034574267
ENSG00000257869.1	5.912242181	4.675980644	1.458865423	3.205217268	0.001349606	0.049213527
ENSG00000258084.6	4.072652597	5.139830914	1.587015706	3.238676779	0.001200856	0.045753727
ENSG00000259075.6	7.385412882	5.982620153	1.606991555	3.722869692	0.000196971	0.013455834

ENSG00000259129.6	47.94418049	3.780888456	0.79097413	4.780040604	1.75E-06	0.000415406
ENSG00000260930.2	3.410204113	4.872780187	1.508168403	3.230925789	0.0012339	0.046346203
ENSG00000262352.2	4.042633981	5.126551905	1.499430019	3.419000447	0.000628516	0.030003922
ENSG00000262576.3	12.41340096	3.7381537	0.96324033	3.880811034	0.000104109	0.008454789
ENSG00000262943.7	66.77385651	2.234003931	0.57251485	3.902089061	9.54E-05	0.008012578
ENSG00000263711.6	15.84677798	7.092755467	1.282654519	5.529747382	3.21E-08	1.68E-05
ENSG00000263745.7	3.77381424	5.020824079	1.477046914	3.399231286	0.000675755	0.031520822
ENSG00000267313.7	91.99826001	2.089513205	0.433346831	4.821803361	1.42E-06	0.000349392
ENSG00000267327.2	55.11746839	3.887291601	0.531184325	7.318159475	2.51E-13	5.69E-10
ENSG00000268658.5	88.36272434	3.833566329	0.468896313	8.175722904	2.94E-16	1.50E-12
ENSG00000271369.1	13.69401803	2.681301995	0.686593068	3.905227304	9.41E-05	0.007988468
ENSG00000276476.3	72.36970937	1.654661549	0.381599212	4.33612412	1.45E-05	0.002205991
ENSG00000276975.3	29.64938448	2.890980892	0.724652693	3.989470984	6.62E-05	0.006183704
ENSG00000279511.1	5.159094591	4.453566839	1.387621196	3.209497559	0.001329672	0.048836088
ENSG00000281344.1	306.630994	2.400004954	0.614687633	3.904430194	9.44E-05	0.007988468
ENSG00000283633.1	39.50730018	3.149844278	0.903362255	3.486800848	0.000488835	0.025681488
ENSG00000285876.1	6.352988119	5.773185509	1.390075873	4.15314417	3.28E-05	0.003798118
ENSG00000286329.1	26.29101883	6.848224936	1.248868002	5.48354584	4.17E-08	1.93E-05
ENSG00000286339.1	135.9737397	7.351382623	0.897825588	8.187985192	2.66E-16	1.50E-12
ENSG00000287248.1	20.51751643	5.563854157	1.115033041	4.989855862	6.04E-07	0.000183834

Down-regulted genes						
**Gene**	**BaseMean**	**log2FoldChange**	**lfcSE**	**stat**	**pvalue**	**padj**
ENSG00000004799.8	157.66943	-3.238477961	0.706369758	-4.584678105	4.55E-06	0.000926833
ENSG00000004948.15	19.90643047	-4.376504698	1.070058972	-4.089965892	4.31E-05	0.004604207
ENSG00000005961.19	120.9677781	-3.706929492	0.520376007	-7.123559577	1.05E-12	2.01E-09
ENSG00000005981.13	81.13974438	-3.79148972	0.738998815	-5.130576182	2.89E-07	0.000107714
ENSG00000008516.17	15.29629468	-4.592208178	1.101268407	-4.169926378	3.05E-05	0.003590153
ENSG00000011422.12	38.62396938	-2.893042931	0.657042449	-4.403129409	1.07E-05	0.001686033
ENSG00000012779.11	34.23300062	-2.839123882	0.729336426	-3.892749327	9.91E-05	0.008196393
ENSG00000018280.17	97.10447862	-4.119115488	0.795667988	-5.1769275	2.26E-07	8.68E-05
ENSG00000022556.16	251.8822545	-3.585415702	0.386766837	-9.270225264	1.86E-20	1.89E-16
ENSG00000025708.14	120.144074	-2.570415405	0.563738557	-4.559587729	5.13E-06	0.000990926
ENSG00000029534.20	120.7097269	-2.011824217	0.477443931	-4.213739223	2.51E-05	0.003180123

ENSG00000047457.14	13.01370452	-3.541765415	1.072121397	-3.303511548	0.00095482	0.039004125
ENSG00000053918.17	53.9905912	-1.60102771	0.40822443	-3.921930174	8.78E-05	0.007602954
ENSG00000064201.15	26.54337729	-3.366209337	0.85553791	-3.934611544	8.33E-05	0.007321666
ENSG00000065911.12	742.4675734	-3.055835865	0.602385333	-5.072892212	3.92E-07	0.000133112
ENSG00000065989.16	22.98544926	-1.842153993	0.466546905	-3.948486154	7.86E-05	0.007031315
ENSG00000070182.21	33.86677719	-2.224736671	0.626524071	-3.550919706	0.000383888	0.021616473
ENSG00000070190.13	10.46732237	-3.582935725	1.089034715	-3.290010572	0.001001836	0.040278952
ENSG00000070669.17	856.9137776	-3.552522339	0.777142183	-4.571264329	4.85E-06	0.00096882
ENSG00000074370.18	97.21439369	-2.345892136	0.462778944	-5.069141897	4.00E-07	0.000133536
ENSG00000074416.14	34.58465163	-2.263305245	0.606019851	-3.734704798	0.000187936	0.013128078
ENSG00000075275.16	177.0974065	-2.120984099	0.582277622	-3.642565021	0.000269935	0.017144916
ENSG00000077454.16	462.0509173	-1.721699103	0.469942634	-3.663636749	0.000248659	0.016004153
ENSG00000078081.8	41.20918085	-2.62825073	0.77223996	-3.403411977	0.000665499	0.031256963
ENSG00000084636.18	404.8092359	-3.537812718	0.845861658	-4.182495665	2.88E-05	0.003477661
ENSG00000085514.16	19.9938559	-3.343406983	0.822034595	-4.067233912	4.76E-05	0.004873157
ENSG00000087074.8	723.6439961	-1.537338491	0.480446827	-3.199809852	0.001375183	0.049526018
ENSG00000087077.14	180.2701547	-1.647072693	0.496155884	-3.319667765	0.000901246	0.03756932
ENSG00000087266.17	1544.195043	-2.165935014	0.623917637	-3.471507911	0.000517544	0.026506579
ENSG00000088827.12	45.61876296	-7.264103743	2.161713281	-3.360345615	0.00077845	0.034159866
ENSG00000089820.15	99.22487945	-2.108248987	0.577539717	-3.650396547	0.000261836	0.016731223
ENSG00000091592.16	54.21150532	-2.498699289	0.669946936	-3.729697315	0.00019171	0.013291888
ENSG00000094755.17	118.835061	-5.325044795	1.02560796	-5.192086065	2.08E-07	8.15E-05
ENSG00000099251.14	26.89751642	-2.761211324	0.73966186	-3.733072464	0.000189158	0.013159726
ENSG00000100055.21	33.58334658	-4.143480883	1.157447592	-3.579843193	0.0003438	0.020372176
ENSG00000100351.17	20.35826616	-3.257636987	0.729741392	-4.464097858	8.04E-06	0.001392841
ENSG00000100368.14	32.03235575	-2.232713542	0.667074589	-3.347022324	0.000816846	0.034980242
ENSG00000100385.14	46.38103785	-2.093126081	0.527032134	-3.97153408	7.14E-05	0.006527568
ENSG00000101162.3	52.92498754	-2.810761216	0.712527759	-3.944774335	7.99E-05	0.007079023
ENSG00000101255.11	761.9099034	-3.128001142	0.770771651	-4.058272172	4.94E-05	0.004972341
ENSG00000101307.15	20.27443178	-2.923270702	0.732150288	-3.992719458	6.53E-05	0.006164257
ENSG00000101670.12	1556.668884	-1.506880484	0.332651171	-4.529911859	5.90E-06	0.001103509
ENSG00000102007.11	56.43865677	-2.223976887	0.638639253	-3.48236798	0.000497	0.025844011
ENSG00000103313.12	23.45362265	-4.827170501	1.144054083	-4.219355163	2.45E-05	0.003121326
ENSG00000103460.17	63.36725448	-2.090060803	0.650476735	-3.213121531	0.001313007	0.048398442

ENSG00000103569.10	12.00361878	-4.513744027	1.024408431	-4.406195705	1.05E-05	0.001675341
ENSG00000105610.5	11.65076336	-4.451025903	0.912354808	-4.878612863	1.07E-06	0.00027566
ENSG00000105641.4	318.7459503	-3.070894454	0.80179139	-3.830041694	0.000128122	0.009968052
ENSG00000105835.12	496.8414967	-1.954654846	0.275888749	-7.084938594	1.39E-12	2.18E-09
ENSG00000105963.15	28.99262026	-1.881317385	0.51243125	-3.671355692	0.000241267	0.015736317
ENSG00000106366.8	136.8850369	-2.72841087	0.662374287	-4.119137661	3.80E-05	0.004212984
ENSG00000106479.11	94.4594435	-1.665551721	0.476215947	-3.497471538	0.000469691	0.025000524
ENSG00000106615.10	228.1456038	-1.55713029	0.445805671	-3.492845405	0.000477903	0.025237245
ENSG00000106617.14	522.4932478	-1.695401552	0.434528758	-3.901701602	9.55E-05	0.008012578
ENSG00000106948.16	1031.120559	-1.83415427	0.545314074	-3.363482363	0.000769658	0.03407203
ENSG00000106952.7	9.285984706	-5.498594723	1.403443259	-3.917931621	8.93E-05	0.007649313
ENSG00000107317.13	34.31511758	-2.247628667	0.691051084	-3.25247832	0.001144033	0.044250424
ENSG00000108405.4	30.6459644	-4.245477247	0.674665339	-6.292715815	3.12E-10	3.17E-07
ENSG00000108556.10	25.74014606	-3.274346596	0.800314028	-4.091327254	4.29E-05	0.004601542
ENSG00000108839.12	5.875736397	-6.348118581	1.463880633	-4.336500148	1.45E-05	0.002205991
ENSG00000108932.12	22.73439254	-4.371498131	1.056185765	-4.138948163	3.49E-05	0.0039077
ENSG00000109321.11	58.39490437	-3.000030006	0.676868723	-4.432218398	9.33E-06	0.001558348
ENSG00000110090.13	593.8083458	-1.667693593	0.400937609	-4.159484063	3.19E-05	0.003736683
ENSG00000110243.11	238.6903354	-1.783129787	0.412050747	-4.327451897	1.51E-05	0.002260889
ENSG00000110324.10	125.1200506	-3.101628614	0.877488962	-3.534663964	0.000408294	0.022372757
ENSG00000110665.11	26.61071063	-3.514884284	0.855623462	-4.107980251	3.99E-05	0.004374168
ENSG00000111371.16	591.2955483	-1.846535872	0.388835103	-4.748891903	2.05E-06	0.000468452
ENSG00000111674.9	57.3893836	-1.979270402	0.55920805	-3.539416864	0.000401012	0.022130054
ENSG00000113739.10	525.4063157	-4.036027471	0.612092828	-6.593815977	4.29E-11	5.46E-08
ENSG00000113763.12	10.62860153	-2.405011231	0.722382738	-3.329275609	0.000870722	0.03674699
ENSG00000114013.16	13.92921282	-3.363743259	0.997859831	-3.37095768	0.000749074	0.03370024
ENSG00000115155.17	27.07986556	-4.129190497	1.158746354	-3.563498157	0.000365945	0.020926996
ENSG00000115165.10	47.2239909	-3.778924476	0.910371745	-4.150968542	3.31E-05	0.003802501
ENSG00000115956.10	82.64201068	-3.864372139	0.838475727	-4.608806211	4.05E-06	0.000851058
ENSG00000116741.8	109.3874778	-1.899760123	0.56748371	-3.347691023	0.000814878	0.034980242
ENSG00000117707.16	204.5926819	-1.561894529	0.446821185	-3.495569553	0.000473051	0.025111132
ENSG00000118523.6	163.5014726	-2.775129949	0.568049778	-4.885364019	1.03E-06	0.000273299
ENSG00000119681.12	33.56695875	-1.516249359	0.46418997	-3.266441453	0.001089083	0.043190414
ENSG00000120055.7	68.77568011	-2.502155891	0.767857275	-3.258621064	0.001119551	0.043894178

ENSG00000120129.6	672.6103099	-2.170016646	0.423421196	-5.124959891	2.98E-07	0.000107714
ENSG00000120875.9	68.74654616	-1.933532105	0.543317982	-3.558748597	0.000372626	0.021124573
ENSG00000120907.18	16.84844849	-3.008878691	0.835072715	-3.60313376	0.000314404	0.019361944
ENSG00000121281.12	273.5126671	-1.533215419	0.384389622	-3.988701393	6.64E-05	0.006183704
ENSG00000121410.12	17.4125629	-2.2747891	0.588785471	-3.863527909	0.000111761	0.008864353
ENSG00000121807.5	6.333873394	-6.45922908	1.628987305	-3.965180734	7.33E-05	0.006614911
ENSG00000122756.15	34.8557423	-2.464751696	0.628652869	-3.920687898	8.83E-05	0.007602954
ENSG00000123405.14	25.2305656	-1.752345974	0.538095852	-3.256568448	0.001127677	0.043894178
ENSG00000124491.15	34.61864998	-3.766320136	0.683600886	-5.509530798	3.60E-08	1.79E-05
ENSG00000124731.13	43.84997746	-4.79353149	1.087717567	-4.406963382	1.05E-05	0.001675341
ENSG00000125740.14	145.3521987	-2.976070033	0.872640257	-3.410420286	0.000648628	0.030819678
ENSG00000125968.9	250.6694569	-1.912755278	0.553406343	-3.456330598	0.000547583	0.027567333
ENSG00000127824.14	31.73823349	-2.254845646	0.659130811	-3.420938012	0.000624056	0.029860911
ENSG00000128965.13	342.6254435	-4.802749863	0.860751807	-5.579715106	2.41E-08	1.33E-05
ENSG00000129911.9	231.6938527	-1.819590076	0.562220488	-3.236435021	0.001210328	0.045916221
ENSG00000129993.15	23.8565499	-2.572053329	0.630140937	-4.081711214	4.47E-05	0.00464688
ENSG00000130294.16	298.8561561	-3.439693107	0.692524585	-4.966889527	6.80E-07	0.000200014
ENSG00000130429.15	393.5713247	-2.007619098	0.401134501	-5.004852722	5.59E-07	0.000172661
ENSG00000130487.9	49.51569977	-4.843036085	0.926637684	-5.226461399	1.73E-07	6.91E-05
ENSG00000131042.14	46.06365756	-5.07129698	1.169143457	-4.337617381	1.44E-05	0.002205991
ENSG00000132334.16	96.63225715	-2.090504055	0.607821572	-3.439338371	0.000583138	0.028437039
ENSG00000133612.19	228.3895986	-1.625037834	0.482236212	-3.369796368	0.000752238	0.03370024
ENSG00000134258.17	36.23685282	-4.426395237	1.101370029	-4.018990096	5.84E-05	0.005619843
ENSG00000134516.17	77.91187698	-3.016831449	0.904711687	-3.334577736	0.00085429	0.036128322
ENSG00000135185.12	128.2446793	-2.031436686	0.352119087	-5.76917514	7.97E-09	5.41E-06
ENSG00000135269.18	377.2194157	-2.616778633	0.542403295	-4.824415072	1.40E-06	0.00034905
ENSG00000135604.10	38.31781284	-2.563344756	0.707570547	-3.622740894	0.000291498	0.018061913
ENSG00000135842.17	604.5889739	-2.336970408	0.670618342	-3.484799416	0.000492506	0.025741652
ENSG00000136327.7	42.14569189	-2.547051955	0.642002263	-3.96735666	7.27E-05	0.006592469
ENSG00000136826.15	95.61319477	-2.548618508	0.573852041	-4.441246746	8.94E-06	0.001519273
ENSG00000136997.19	67.02470769	-1.802385925	0.477485585	-3.774744165	0.000160172	0.01161655
ENSG00000137285.10	184.8687983	-2.118568569	0.439102377	-4.824771356	1.40E-06	0.00034905
ENSG00000137331.12	76.35591617	-1.884907752	0.560240956	-3.364459047	0.000766939	0.03407203
ENSG00000137801.11	1609.581844	-3.349277885	0.724496427	-4.622904628	3.78E-06	0.000803477

ENSG00000137959.16	104.5769459	-4.99687681	1.057856595	-4.723586195	2.32E-06	0.000519057
ENSG00000137965.11	31.34452874	-4.69079975	1.279587731	-3.665868025	0.000246501	0.015951356
ENSG00000139269.3	108.4357643	-2.528652671	0.530393342	-4.767504548	1.87E-06	0.000437018
ENSG00000139514.13	621.8365975	-2.465511993	0.539287457	-4.571795544	4.84E-06	0.00096882
ENSG00000140379.8	12.42392634	-4.041381883	1.033053207	-3.912075249	9.15E-05	0.007804461
ENSG00000140678.16	83.13413532	-4.465162322	1.077355427	-4.14455825	3.40E-05	0.003855627
ENSG00000141506.13	60.06123852	-3.192441737	0.923276469	-3.457731076	0.000544745	0.027553546
ENSG00000141582.15	339.9341965	-2.628147412	0.619419502	-4.24292003	2.21E-05	0.002864537
ENSG00000141682.11	52.03571743	-1.853498334	0.550334062	-3.367951326	0.00075729	0.033852176
ENSG00000141968.8	17.37041209	-2.840830631	0.79678856	-3.565350677	0.00036337	0.020926996
ENSG00000142102.16	122.718893	-2.247142116	0.597468225	-3.761107325	0.000169163	0.012099
ENSG00000142185.16	27.97122645	-3.5276108	0.798271399	-4.419061993	9.91E-06	0.001616536
ENSG00000142552.8	204.7267853	-1.957448144	0.537625813	-3.64091176	0.000271674	0.017144916
ENSG00000143632.14	11.91585656	-3.130916203	0.87870725	-3.563093629	0.00036651	0.020926996
ENSG00000145423.5	36.37496932	-3.530813387	1.094833729	-3.224976811	0.001259829	0.04686195
ENSG00000146112.12	387.5877873	-1.5688424	0.47380354	-3.311166482	0.000929079	0.038372637
ENSG00000146592.17	23.70176136	-1.843562732	0.485016925	-3.801027628	0.000144097	0.010838664
ENSG00000146678.10	9.171392425	-5.474414561	1.617027363	-3.385480473	0.000710538	0.032484926
ENSG00000147872.10	1695.173847	-4.052433908	0.803152963	-5.045656424	4.52E-07	0.000143951
ENSG00000148339.12	508.094609	-1.870405243	0.567061881	-3.2984147	0.000972324	0.039430443
ENSG00000148408.13	65.68158001	-1.752002292	0.45984832	-3.809956924	0.000138991	0.01073179
ENSG00000148488.17	19.76836002	-4.086656439	1.137510842	-3.59263076	0.000327356	0.019800685
ENSG00000148908.15	40.20376055	-2.379948084	0.423919459	-5.614151539	1.98E-08	1.18E-05
ENSG00000149781.12	73.96998725	-2.542588621	0.75601248	-3.363156943	0.000770565	0.03407203
ENSG00000150594.7	17.60253204	-2.569270046	0.637815434	-4.02823436	5.62E-05	0.005480985
ENSG00000150681.10	14.4774392	-4.225026605	1.134549114	-3.723969772	0.000196114	0.013455834
ENSG00000151650.8	3.386858539	-5.535659895	1.67837734	-3.298221302	0.000972994	0.039430443
ENSG00000151952.16	12.32466143	-2.218589862	0.612409962	-3.622720071	0.000291521	0.018061913
ENSG00000153404.14	31.22843419	-1.989873118	0.57798014	-3.442805346	0.000575714	0.028437039
ENSG00000154118.13	70.6234753	-1.544781639	0.378671653	-4.079475253	4.51E-05	0.00464688
ENSG00000154451.14	26.06739748	-4.249932622	1.074668965	-3.954643486	7.66E-05	0.006882865
ENSG00000155307.18	17.62737367	-3.528380834	1.025838786	-3.439508119	0.000582772	0.028437039
ENSG00000155897.10	9.924189264	-3.668045556	1.046261185	-3.505860303	0.000455134	0.024478765
ENSG00000158764.7	97.87916906	-1.950156302	0.427382506	-4.563023227	5.04E-06	0.000988276

ENSG00000159399.9	1318.570516	-2.094073321	0.476812813	-4.391814282	1.12E-05	0.001762568
ENSG00000159784.17	14.01362385	-2.815497949	0.673693843	-4.179195012	2.93E-05	0.00350776
ENSG00000159840.16	665.6208847	-1.588408257	0.43358827	-3.663402277	0.000248887	0.016004153
ENSG00000160352.16	256.3641983	-2.899298383	0.464983068	-6.235277331	4.51E-10	4.18E-07
ENSG00000160360.13	305.5771751	-2.495065748	0.771665175	-3.23335279	0.001223464	0.046098123
ENSG00000160469.17	188.2701119	-2.258422336	0.680233532	-3.320069108	0.000899952	0.03756932
ENSG00000160813.7	79.17472544	-2.128468351	0.651488915	-3.267082987	0.001086618	0.043176659
ENSG00000160991.16	93.94165429	-1.859536954	0.300437707	-6.189425995	6.04E-10	5.35E-07
ENSG00000162367.11	55.65908407	-1.649874515	0.40732698	-4.050491608	5.11E-05	0.005057423
ENSG00000162511.8	290.2502032	-2.656230401	0.744443002	-3.5680776	0.00035961	0.020870055
ENSG00000162711.17	40.55284844	-4.364779665	1.23042004	-3.547389933	0.000389068	0.021787265
ENSG00000162804.14	14.46840973	-2.044287247	0.61692018	-3.31369813	0.000920709	0.038145777
ENSG00000163734.4	6.469900097	-3.271751066	0.972270344	-3.365063108	0.000765262	0.03407203
ENSG00000163736.4	19.65740612	-4.327283492	0.947284063	-4.568094893	4.92E-06	0.000974033
ENSG00000163737.4	43.83968582	-2.71341248	0.777219594	-3.491178685	0.000480895	0.025329596
ENSG00000164406.8	803.3735542	-1.863814016	0.529488807	-3.520025338	0.000431506	0.023455421
ENSG00000164707.15	7.29871653	-3.792165737	1.147290075	-3.305324275	0.000948666	0.038908656
ENSG00000164897.14	233.7962487	-1.742529934	0.501636714	-3.473688994	0.000513356	0.026424854
ENSG00000165125.20	86.19697368	-2.350752574	0.479026187	-4.907357134	9.23E-07	0.000247635
ENSG00000165702.14	15.95949732	-2.402296421	0.701200753	-3.425975242	0.000612596	0.029417806
ENSG00000165802.22	263.3043961	-1.776829936	0.497689396	-3.570158314	0.000356766	0.020870055
ENSG00000166091.21	4.54742399	-3.821448046	1.173520706	-3.256395925	0.001128363	0.043894178
ENSG00000166501.14	82.24444717	-2.326060545	0.629531186	-3.69490916	0.000219965	0.014557711
ENSG00000167207.13	19.26843348	-1.921684803	0.552571122	-3.47771486	0.000505708	0.026097071
ENSG00000167208.15	12.71994216	-4.330112575	1.202414987	-3.60117981	0.000316776	0.0193909
ENSG00000167772.12	252.4208021	-1.582221707	0.487356478	-3.246538785	0.001168175	0.044928463
ENSG00000167851.15	19.79927692	-2.990647855	0.88288769	-3.387348005	0.000705718	0.032399454
ENSG00000168209.5	2307.375545	-3.42183175	0.589412614	-5.805494603	6.42E-09	4.51E-06
ENSG00000168229.4	3.372146001	-4.752540564	1.479085639	-3.213161185	0.001312826	0.048398442
ENSG00000168268.11	752.6568114	-1.526659173	0.2677594	-5.701608136	1.19E-08	7.56E-06
ENSG00000169508.7	29.69815441	-2.779044274	0.80362673	-3.458128222	0.000543942	0.027553546
ENSG00000169704.4	3.527897582	-5.601699689	1.506719478	-3.717811955	0.000200956	0.013608911
ENSG00000170345.10	463.1077342	-3.481380684	0.828123193	-4.203940567	2.62E-05	0.003260295
ENSG00000171466.10	112.8983069	-2.902473734	0.370431819	-7.835379095	4.67E-15	1.59E-11

ENSG00000171502.15	14.75982863	-3.399773181	0.671843035	-5.060368279	4.18E-07	0.000137575
ENSG00000171522.6	28.24571447	-1.613542693	0.503015527	-3.207739337	0.001337827	0.048942961
ENSG00000171860.5	10.93886091	-3.260915969	0.990318938	-3.29279371	0.000991972	0.040040323
ENSG00000172059.11	134.6763623	-2.344653739	0.475432758	-4.931620089	8.16E-07	0.000224638
ENSG00000172159.16	26.21982586	-1.861542909	0.491316441	-3.788887878	0.000151323	0.011251318
ENSG00000172216.6	321.2450408	-1.665710399	0.49582586	-3.359466567	0.000780931	0.034159866
ENSG00000172243.17	47.75279887	-4.654060804	1.041238601	-4.469735178	7.83E-06	0.00137621
ENSG00000172322.14	14.36252372	-4.744050168	1.457470128	-3.254989641	0.001133965	0.044028082
ENSG00000172354.10	841.6650728	-1.50789814	0.438079665	-3.442063765	0.000577294	0.028437039
ENSG00000172432.19	538.0270519	-2.113000065	0.660277835	-3.200168102	0.001373475	0.049526018
ENSG00000173110.8	21.82817556	-2.984654847	0.822898244	-3.627003545	0.000286729	0.017873674
ENSG00000173237.4	278.4388645	-3.518198691	0.820942693	-4.285559418	1.82E-05	0.002562479
ENSG00000174672.16	90.69252184	-2.542107915	0.65781601	-3.864466474	0.000111332	0.008864353
ENSG00000175084.12	17.03328951	-2.548628166	0.735349431	-3.465873579	0.000528512	0.027000459
ENSG00000175592.9	5.766516924	-3.346529963	1.035987064	-3.230281612	0.001236683	0.046346203
ENSG00000176845.13	121.7209718	-1.54375616	0.371455737	-4.155962629	3.24E-05	0.003773029
ENSG00000177989.13	16.24250856	-2.728836056	0.66401742	-4.10958504	3.96E-05	0.004367363
ENSG00000178562.18	10.88242399	-5.170600358	1.248271683	-4.142207526	3.44E-05	0.003873848
ENSG00000182648.13	44.92399934	-2.138841777	0.384327211	-5.565158325	2.62E-08	1.40E-05
ENSG00000183010.17	684.2574317	-5.25876478	0.688768693	-7.635022947	2.26E-14	5.75E-11
ENSG00000183439.9	17.65753736	-4.880261602	0.857871001	-5.688805887	1.28E-08	7.90E-06
ENSG00000184261.4	101.4132382	-1.824556053	0.564056519	-3.234704311	0.001217688	0.045965454
ENSG00000185215.9	87.14675461	-2.06112807	0.61429549	-3.355271368	0.000792872	0.034241305
ENSG00000185245.8	9.938928671	-4.599995647	1.028821878	-4.471129305	7.78E-06	0.00137621
ENSG00000185561.10	257.1789387	-1.847371509	0.518655978	-3.561843661	0.00036826	0.02096817
ENSG00000185862.7	48.45842685	-3.903540773	1.208611689	-3.229772481	0.001238888	0.046346203
ENSG00000185885.16	62.6960419	-2.363058696	0.539848548	-4.377262296	1.20E-05	0.001870027
ENSG00000185950.9	472.0653353	-2.144449922	0.396299477	-5.411185342	6.26E-08	2.83E-05
ENSG00000186407.7	54.86680946	-3.743562959	1.08535784	-3.449150889	0.000562352	0.028026872
ENSG00000187840.5	577.0744147	-1.926644188	0.591603887	-3.256645585	0.001127371	0.043894178
ENSG00000188536.13	218.542599	-4.142303822	0.864937586	-4.789136106	1.68E-06	0.000401687
ENSG00000196411.10	3149.853533	-1.869705822	0.467892023	-3.99601987	6.44E-05	0.006107267
ENSG00000196453.8	132.4269008	-1.785305537	0.419154428	-4.259302583	2.05E-05	0.002768251
ENSG00000196517.11	302.2898959	-2.435271598	0.698973321	-3.484069455	0.000493851	0.025745947

ENSG00000197405.8	94.18097152	-3.924169079	0.870098839	-4.510026793	6.48E-06	0.001179714
ENSG00000197632.9	8.141548874	-6.803355625	1.631514425	-4.169963515	3.05E-05	0.003590153
ENSG00000197993.9	14.47812867	-2.43900869	0.570294796	-4.276750742	1.90E-05	0.002647699
ENSG00000198650.11	441.9556545	-2.830805273	0.660246594	-4.28749697	1.81E-05	0.002557877
ENSG00000204054.14	206.5771985	-2.024514783	0.498908493	-4.057887991	4.95E-05	0.004972341
ENSG00000204099.11	248.4882707	-2.38217348	0.582631832	-4.088642859	4.34E-05	0.004604207
ENSG00000204389.10	862.3668793	-2.709357972	0.765578394	-3.538968697	0.000401693	0.022130054
ENSG00000204420.10	29.16039546	-4.274860329	0.791793903	-5.398955856	6.70E-08	2.91E-05
ENSG00000204424.9	5.383508968	-6.219159679	1.354303108	-4.592147537	4.39E-06	0.000903295
ENSG00000204482.10	10.41842468	-4.656165104	1.338514008	-3.478607677	0.000504026	0.026076304
ENSG00000204983.14	43.10381029	-3.788084913	0.739609673	-5.121735219	3.03E-07	0.000107714
ENSG00000205710.4	216.6412692	-2.299139464	0.563435104	-4.080575469	4.49E-05	0.00464688
ENSG00000205795.4	13.652558	-2.071909425	0.634580359	-3.265007176	0.001094613	0.043325421
ENSG00000206172.8	6.632532217	-5.775556146	1.407832839	-4.102444543	4.09E-05	0.004456224
ENSG00000206190.11	11.19586725	-2.540382676	0.752003956	-3.378150681	0.000729751	0.033129712
ENSG00000206432.4	37.58597079	-1.726056997	0.455649971	-3.788120502	0.000151791	0.011251318
ENSG00000215146.5	4.781812735	-6.053162711	1.422882976	-4.254153583	2.10E-05	0.002795689
ENSG00000215218.4	28.28047598	-2.044260336	0.569255532	-3.591111936	0.00032927	0.019857524
ENSG00000223756.6	6.664209722	-5.781746174	1.336731569	-4.325285874	1.52E-05	0.002266555
ENSG00000223802.7	65.44886505	-1.735987503	0.534910981	-3.245376451	0.001172955	0.04497814
ENSG00000224397.7	12.32527531	-4.600507723	1.227010043	-3.749364358	0.000177283	0.012547722
ENSG00000225968.7	205.6490037	-1.557915913	0.420548289	-3.704487576	0.000211818	0.01415642
ENSG00000229644.6	35.13397667	-2.799755312	0.52395549	-5.343498383	9.12E-08	3.79E-05
ENSG00000233297.4	9.800841252	-3.701931099	1.037680049	-3.567507252	0.000360393	0.020870055
ENSG00000235568.6	28.81836829	-3.335619218	1.034251557	-3.225152714	0.001259055	0.04686195
ENSG00000236404.10	8.846034963	-3.823272806	0.981961174	-3.893507103	9.88E-05	0.008196393
ENSG00000239911.2	43.6091832	-1.96832248	0.551606589	-3.568344756	0.000359244	0.020870055
ENSG00000241685.10	451.4934219	-1.546644807	0.329058547	-4.70021162	2.60E-06	0.000569639
ENSG00000248323.7	19.79903214	-5.609600252	1.264537558	-4.43608829	9.16E-06	0.001543259
ENSG00000249364.6	41.80401913	-1.525379739	0.461324899	-3.306519426	0.000944628	0.038821164
ENSG00000255197.6	12.76246679	-3.862475755	1.14443269	-3.375013479	0.000738121	0.033358004
ENSG00000259171.1	109.3107567	-1.603477351	0.392473135	-4.085572253	4.40E-05	0.004619846
ENSG00000259207.7	156.6746632	-2.214024925	0.446649924	-4.956958022	7.16E-07	0.000205578
ENSG00000259803.7	11.10877206	-3.479423335	1.041056765	-3.34220328	0.000831162	0.0354367

ENSG00000260997.1	9.517178978	-4.961760683	1.245323299	-3.984315308	6.77E-05	0.006270393
ENSG00000261455.1	15.8299009	-2.372628193	0.628187727	-3.776941335	0.000158766	0.01161655
ENSG00000262001.1	13.71591457	-2.911445984	0.818275755	-3.558025478	0.000373653	0.021124573
ENSG00000262227.1	21.43179954	-2.574492327	0.718772463	-3.581790424	0.000341248	0.020279851
ENSG00000265206.5	6.82773486	-4.411102486	1.275051923	-3.459547337	0.000541084	0.027553546
ENSG00000267368.1	101.3138259	-3.044279201	0.829839387	-3.668516161	0.000243962	0.015837347
ENSG00000267519.6	191.5851685	-3.58002196	0.580902049	-6.162866812	7.14E-10	6.07E-07
ENSG00000267943.1	10.23360139	-3.769349657	0.947559429	-3.977955939	6.95E-05	0.006411298
ENSG00000272079.2	15.70883553	-2.97373297	0.787040816	-3.778371983	0.000157857	0.01161655
ENSG00000272398.6	1314.837397	-2.172748387	0.558659425	-3.889218173	0.000100568	0.008266014
ENSG00000272512.1	8.759756264	-2.357511883	0.710166281	-3.319661811	0.000901266	0.03756932
ENSG00000274750.2	43.77771003	-6.470695922	0.839040573	-7.712017909	1.24E-14	3.61E-11
ENSG00000275620.1	14.31714038	-2.901739562	0.808423046	-3.589382535	0.000331462	0.01993075
ENSG00000275896.5	11.34639527	-6.565185698	1.280749371	-5.126050302	2.96E-07	0.000107714
ENSG00000276107.1	5.083948984	-5.362369147	1.429462717	-3.751317947	0.000175907	0.01249372
ENSG00000276805.2	53.16804457	-1.800974449	0.495742155	-3.632885422	0.000280269	0.017524581
ENSG00000280407.2	7.176707138	-3.212717918	0.93081201	-3.451521773	0.000557435	0.027918306
ENSG00000284707.2	45.14085695	-2.947412712	0.877513315	-3.358823918	0.000782749	0.034166083
