# Supplementary material for: Human-induced pluripotent stem cell–based hepatic modeling of lipid metabolism–associated TM6SF2-E167K variant
Source: Hepatology. Author manuscript; Available in PMC 2025 Aug 23. (PMC11865362; doi:10.1097/HEP.0000000000001065)
Supplement: Supplementary file 10 [file NIHMS2022610-supplement-Sup_Tab_5.pdf]

Supplementary table 5a: Characteristics of healthy cohorts analyzed.

Count in Group	Group	Age (Years)	Gender	Ethnicity	PNPLA3 Genotype	MBOAT7 Genotype	GCKR Genotype	TM6SF2 Genotype
1	Healthy	65	Male	Hispanic American	CC	CT	CC	CC
2	Healthy	60	Female	White American	CG	TT	CT	CC
3	Healthy	47	Female	White American	CC	CC	CC	CC
4	Healthy	22	Male	White American	CC	TT	CT	CC
5	Healthy	64	Female	Hispanic American	GG	CT	CT	CC
6	Healthy	15	Male	White American	CC	CT	CT	CC
7	Healthy	17	Male	White American	CC	CT	CT	CC
8	Healthy	21	Female	White American	CG	TT	CC	CC
9	Healthy	59	Female	White American	CC	CC	TT	CC
10	Healthy	51	Male	White American	CC	CC	CT	CC
11	Healthy	42	Male	Hispanic American	CC	CC	TT	CC
12	Healthy	68	Female	White American	CC	CC	CC	CC
13	Healthy	40	Female	White American	CG	CT	CC	CC
14	Healthy	49	Male	White American	CC	TT	CT	CC
15	Healthy	46	Female	White American	GG	TT	TT	CC
16	Healthy	55	Male	White American	CC	CC	CC	CC
17	Healthy	45	Male	White American	CC	CC	CT	CC
18	Healthy	69	Female	White American	CC	TT	CC	CC
19	Healthy	53	Male	White American	CC	CT	CT	CC
20	Healthy	57	Male	White American	CC	CT	CT	CC
21	Healthy	37	Male	Hispanic American	GG	CT	TT	CC
22	Healthy	67	Male	White American	CG	CC	CC	CC
23	Healthy	9	Male	Hispanic American	CC	TT	TT	CC
24	Healthy	45	Male	Hispanic American	CG	CT	CT	CC
25	Healthy	44	Male	Hispanic American	CG	TT	CC	CC
26	Healthy	50	Male	White American	CC	TT	CT	CC
27	Healthy	23	Male	White American	CC	CC	CT	CC
28	Healthy	44	Male	White American	CC	CC	TT	CC
29	Healthy	33	Female	White American	CC	CT	CC	CC
30	Healthy	52	Female	White American	CC	CT	CC	CC
31	Healthy	17	Male	Black or African America	CG	TT	CC	CC
32	Healthy	65	Male	White American	CC	CT	CC	CC
33	Healthy	18	Female	White American	CG	TT	CC	CC
34	Healthy	49	Male	White American	CC	CC	CT	CT
35	Healthy	55	Female	White American	CC	CT	CT	CC
36	Healthy	61	Female	Black or African America	CG	CT	CT	CC
37	Healthy	18	Male	Hispanic American	CG	CC	TT	CC
38	Healthy	40	Female	White American	GG	TT	CT	TT
39	Healthy	34	Male	White American	CC	CT	CT	CC
40	Healthy	41	Female	White American	CC	CC	CC	CT
41	Healthy	53	Female	White American	CG	TT	CC	CC
42	Healthy	66	Female	White American	CC	CT	CT	CC
43	Healthy	48	Female	Black or African America	CC	CT	CC	CC
44	Healthy	45	Female	White American	CG	TT	CT	CC
45	Healthy	33	Male	White American	CG	CT	CT	CC
46	Healthy	77	Female	Hispanic American	CG	CC	CC	CC
47	Healthy	44	Female	White American	CC	CT	CC	CT
48	Healthy	48	Male	White American	CG	CT	CT	CC
49	Healthy	57	Female	White American	CC	CT	CT	CC
50	Healthy	4	Female	Hispanic American	CG	CC	CC	CC
51	Healthy	40	Male	White American	CC	TT	CC	CC
52	Healthy	62	Female	White American	CG	CT	CT	CC
53	Healthy	41	Male	Black or African America	CC	CC	CT	CC
54	Healthy	43	Female	Hispanic American	GG	CC	TT	CC
55	Healthy	46	Male	White American	CG	CT	CT	CC
56	Healthy	57	Male	White American	CC	CT	CC	CC
57	Healthy	19	Female	Black or African America	CC	CT	CT	CC
58	Healthy	44	Female	Other	CG	CC	CC	CC
59	Healthy	62	Female	Hispanic American	CC	CC	TT	CT
60	Healthy	59	Female	Other	CG	CT	CC	CC
61	Healthy	44	Female	Hispanic American	CG	CC	CC	CC
62	Healthy	27	Male	White American	CC	CT	CC	CC

63	Healthy	53	Female	White American	CC	CC	CT	CC
64	Healthy	47	Male	Hispanic American	GG	CC	CT	CC
65	Healthy	52	Female	White American	GG	CT	CT	CC
66	Healthy	25	Male	White American	CG	CC	CT	CC
67	Healthy	54	Male	White American	CC	CT	CC	CC
68	Healthy	31	Male	White American	CG	CC	CT	CC
69	Healthy	44	Male	Hispanic American	CG	CC	CT	CC
70	Healthy	64	Female	White American	CG	CC	CT	CC
71	Healthy	24	Female	Hispanic American	CG	CT	CT	CC
72	Healthy	33	Female	White American	CG	TT	TT	CC
73	Healthy	24	Male	Black or African America	CG	CT	CC	CC
74	Healthy	61	Female	White American	CG	CT	CT	CC
75	Healthy	41	Male	White American	CC	CT	CC	CC
76	Healthy	40	Male	White American	CG	CT	CT	CC
77	Healthy	43	Male	White American	CC	CT	CC	CC
78	Healthy	38	Male	White American	CC	CT	CT	CC
79	Healthy	58	Female	NO DATA	CC	CT	CT	CC
80	Healthy	46	Female	Hispanic American	CG	CT	CC	CC
81	Healthy	1	Male	White American	CC	CC	CT	CC
82	Healthy	57	Female	White American	CG	TT	TT	CC
83	Healthy	58	Male	White American	CC	TT	CC	CC
84	Healthy	45	Female	Other	CG	TT	TT	CC
85	Healthy	27	Female	White American	GG	CC	CT	CC
86	Healthy	65	Female	Hispanic American	CC	CC	CC	CC
87	Healthy	24	Female	White American	CG	TT	CT	CC
88	Healthy	26	Female	White American	CC	CC	CT	CT
89	Healthy	66	Male	White American	CG	TT	CC	CC
90	Healthy	71	Female	White American	CC	CT	CT	CC
91	Healthy	52	Female	White American	CC	CT	CT	CC
92	Healthy	37	Female	White American	CC	CT	CT	CC
93	Healthy	66	Female	Hispanic American	CC	CT	CC	CC
94	Healthy	35	Male	White American	CC	CT	CT	CC
95	Healthy	45	Male	Black or African America	CC	TT	CC	CT
96	Healthy	26	Female	White American	CG	CC	TT	CC
97	Healthy	55	Female	White American	CG	TT	CC	CC
98	Healthy	69	Female	Hispanic American	CG	TT	CT	CC
99	Healthy	52	Male	White American	CC	CC	CC	CC
100	Healthy	53	Female	White American	CC	CT	CT	CT
101	Healthy	35	Male	Black or African America	CC	CT	CC	CC
102	Healthy	28	Male	White American	CG	CT	CC	CC
103	Healthy	42	Male	White American	CG	CT	CT	CC
104	Healthy	39	Female	Hispanic American	CC	CC	CC	CC
105	Healthy	51	Male	White American	CC	CT	CC	CC
106	Healthy	53	Female	White American	CG	TT	CC	CC
107	Healthy	15	Male	White American	CG	CT	CC	CC
108	Healthy	67	Male	White American	CG	TT	TT	CC
109	Healthy	27	Female	White American	CG	CC	CT	CT
110	Healthy	56	Female	White American	CC	CT	CC	CC
111	Healthy	56	Male	White American	CC	TT	CT	CC
112	Healthy	17	Male	White American	CC	CT	CT	CC
113	Healthy	35	Male	White American	CC	CC	CC	CC
114	Healthy	49	Female	Other	CG	CT	CT	CC
115	Healthy	57	Male	White American	CC	CT	CC	CC
116	Healthy	66	Female	White American	CC	CT	CC	CC
117	Healthy	59	Male	White American	CC	CT	CC	CC
118	Healthy	64	Female	White American	CG	CT	TT	CC
119	Healthy	48	Male	White American	CC	CT	CT	CC
120	Healthy	57	Male	Black or African America	CC	CT	CT	CC
121	Healthy	62	Male	Black or African America	CC	CC	CT	CC
122	Healthy	58	Male	White American	CC	CT	CC	CC
123	Healthy	71	Female	White American	CC	CC	CC	CT

Supplementary table 5b: Characteristics of ESLD cohorts analyzed.

Count in Group	Group1	Age (Years)	Sex1	PNPLA3 Genotype	MBOAT7 Genotype	GCKR Genotype	TM6SF2 Genotype
1	ESLD	64	Female	GG	TT	CT	CC
2	ESLD	53	Male	GG	TT	TT	CC
3	ESLD	68	Female	GG	CT	CT	CT
4	ESLD	71	Female	CC	CC	CT	CC
5	ESLD	71	Female	GG	CC	CT	CC
6	ESLD	65	Male	CG	CC	CC	CC
7	ESLD	50	Female	CC	TT	CT	CC
8	ESLD	63	Male	GG	CC	CT	CC
9	ESLD	64	Female	CG	TT	CC	CT
10	ESLD	63	Female	CC	CC	CC	CC
11	ESLD	67	Male	CC	CC	TT	CC
12	ESLD	60	Male	CG	CT	TT	CC
13	ESLD	48	Female	GG	CT	TT	CC
14	ESLD	58	Male	GG	CC	CT	CC
15	ESLD	69	Female	CC	CT	CT	CC
16	ESLD	57	Female	CG	CT	CT	CC
17	ESLD	71	Female	GG	CT	CC	CC
18	ESLD	54	Female	GG	CT	CC	CC
19	ESLD	64	Female	CG	CC	CT	CC
20	ESLD	62	Female	GG	TT	CC	CC
21	ESLD	70	Male	CG	TT	CT	CT
22	ESLD	62	Female	GG	CT	CT	CC
23	ESLD	59	Male	CG	CT	CT	CC
24	ESLD	67	Female	CC	TT	CT	CC
25	ESLD	63	Male	CG	CT	CT	CC
26	ESLD	52	Female	CG	TT	TT	CC
27	ESLD	54	Male	CC	TT	CT	CC
28	ESLD	58	Male	CC	CT	CT	CC
29	ESLD	70	Female	GG	CC	TT	CC
30	ESLD	59	Male	CG	CT	TT	CT
31	ESLD	58	Male	CG	TT	CC	CC
32	ESLD	63	Male	GG	CT	CT	CT
33	ESLD	61	Female	CG	CT	TT	CC
34	ESLD	62	Female	CG	CT	CT	CT
35	ESLD	56	Male	CC	TT	CC	CT
36	ESLD	66	Male	GG	CT	CT	CC
37	ESLD	65	Female	CG	TT	CC	CC
38	ESLD	57	Male	GG	TT	CT	CC
39	ESLD	69	Female	GG	CC	CC	CC
40	ESLD	57	Male	CG	CT	TT	CC
41	ESLD	61	Female	CG	CT	CC	CC
42	ESLD	55	Female	CG	CT	CC	CC
43	ESLD	54	Male	GG	CT	CT	CC
44	ESLD	61	Male	GG	TT	CC	TT
45	ESLD	72	Female	CG	TT	CC	CC
46	ESLD	72	Female	CG	TT	CC	CC
47	ESLD	72	Female	CC	CC	CT	CC
48	ESLD	55	Male	CG	CC	CT	CT
49	ESLD	49	Male	CG	CC	TT	CC
50	ESLD	74	Male	GG	CT	CT	CT
